# Identification of an autotransporter peptidase of *Rickettsia rickettsii* responsible for maturation of surface exposed autotransporters

**DOI:** 10.1371/journal.ppat.1011527

**Published:** 2023-07-31

**Authors:** Adam M. Nock, Karin Aistleitner, Tina R. Clark, Dan Sturdevant, Stacy Ricklefs, Kimmo Virtaneva, Yixiang Zhang, Naila Gulzar, Neelam Redekar, Amitiva Roy, Ted Hackstadt

**Affiliations:** 1 Host-Parasite Interactions Section, Laboratory of Bacteriology, Rocky Mountain Laboratories, NIAID, NIH; Hamilton, Montana, United States of America; 2 Genomics Research Section, Research Technologies Branch, Rocky Mountain Laboratories, NIAID, NIH; Hamilton, Montana, United States of America; 3 Protein Chemistry Unit, Research Technologies Branch, Rocky Mountain Laboratories, NIAID, NIH; Hamilton, Montana, United States of America; 4 Integrated Data Sciences Section, Research Technologies Branch, NIAID, NIH, Bethesda, Maryland, United States of America; 5 Bioinformatics and Computational Biology Branch, Office of Cyber Infrastructure and Computational Biology, Rocky Mountain Laboratories, NIAID, NIH; Hamilton, Montana, United States of America; Northwestern University Feinberg School of Medicine, UNITED STATES

## Abstract

Members of the spotted fever group rickettsia express four large, surface-exposed autotransporters, at least one of which is a known virulence determinant. Autotransporter translocation to the bacterial outer surface, also known as type V secretion, involves formation of a β-barrel autotransporter domain in the periplasm that inserts into the outer membrane to form a pore through which the N-terminal passenger domain is passed and exposed on the outer surface. Two major surface antigens of *Rickettsia rickettsii*, are known to be surface exposed and the passenger domain cleaved from the autotransporter domain. A highly passaged strain of *R*. *rickettsii*, Iowa, fails to cleave these autotransporters and is avirulent. We have identified a putative peptidase, truncated in the Iowa strain, that when reconstituted into Iowa restores appropriate processing of the autotransporters as well as restoring a modest degree of virulence.

## Author summary

Strains of *R*. *rickettsii*, the agent of Rocky Mountain spotted fever, differ greatly in the severity of the disease caused. The genetic differences responsible for this disparity are only now being uncovered. An avirulent, laboratory adapted strain of *R*. *rickettsii* fails to proteolytically process several large surface protein antigens. We have identified a protease that cleaves the protein precursors to their mature form. The gene encoding this protease is mutated in the avirulent strain. Complementation of the active form of the gene identifies proteolytic processing of surface antigens as important to virulence.

## Introduction

Members of the genus *Rickettsia* are arthropod-borne obligate intracellular bacteria responsible for various spotted fever rickettsioses around the world. Rickettsiae are classified into the spotted fever group (SFG), typhus group, ancestral group, or transitional group [1]. Among the SFG is *R*. *rickettsii*, the agent of Rocky Mountain spotted fever, which has the highest mortality rate of any rickettsioses. Other species within the SFG cause less severe disease or are not associated with disease in humans and may be simply endosymbionts within ticks. Transovarial transmission is common in SFG rickettsiae and horizonal transmission may not be an essential part of their natural history, particularly among the nonpathogenic species.

Within the species *R*. *rickettsii*, there is considerable variation between isolates in the severity of disease caused. This is reflected both in humans, where great differences in mortality are observed within relatively close geographic regions [2], and in animals, in which fever responses are dependent upon the infecting strain [3–7]. The genetic bases for these differences are only now beginning to be understood. Some time ago, the failure of the avirulent Iowa strain of *R*. *rickettsii* to proteolytically process a major surface antigen was noted [8,9]. The protein, rOmpB, is a type V secreted or autotransporter protein [10]. Follow-up studies confirmed the failure of a lower passage Iowa strain to correctly process rOmpA, another large autotransporter and surface antigen [11]. The amino acid sequence near the cleavage site was very similar in both proteins. SFG rickettsiae encode at least four surface-exposed autotransporters [12]. Indeed, all four SFG autotransporters, rOmpA, rOmpB, Sca1, and Sca2 displayed similar sequences near the known or predicted cleavage site [11].

Autotransporters represent one of several mechanisms used by Gram-negative bacteria to secrete virulence factors to the bacterial outer surface or release into the external environment [13]. Autotransporters consist of three domains, an N-terminal secretory signal, a larger, N-terminal passenger domain, and a C-terminal β-barrel domain that facilitates the translocation of the passenger domain to the extracellular face of the bacterial outer membrane [14,15]. Autotransporters may remain intact as a large protein exposed on the outer surface of the bacterium but are frequently post-translationally processed by proteolysis subsequent to surface translocation. This proteolytic processing may be by autocatalysis or by a unique protease to leave a membrane associated β fragment and a passenger domain which may remain non-covalently associated with the β fragment or be released in a soluble form [10,15].

An initial genomic comparison of the highly virulent *R*. *rickettsii* Sheila Smith strain to the laboratory attenuated Iowa strain identified 492 single nucleotide polymorphisms (SNPs) and 143 insertions/deletions (InDels) [6]. Genomic sequencing of additional strains revealed a close relationship between the Sheila Smith and R strains as well as a close relationship between the virulent Morgan strain and Iowa strain [5]. Further analysis identified a limited number of mutations unique to Iowa but failed to explain the inability of Iowa to process rOmpA or rOmpB. This study did reveal, however, that rOmpB from Iowa and Morgan were identical, thus it is unlikely that this processing is autocatalytic.

To extend the search for a putative autotransporter peptidase, we considered that mutations other than directly to the protein itself might be involved. We thus initiated an RNAseq experiment to compare the transcriptomes of Morgan and Iowa. In this manner, we identified a gene mutated in Iowa that, when expressed as the full-length wild-type gene in Iowa, restores cleavage of rOmpB and rOmpA. This peptidase is predicted to be a lipoprotein. We have named this autotransporter peptidase Rickettsial autotransporter peptidase Lipoprotein (RapL).

## Results

### Comparison of the transcriptome of Iowa and Morgan strains

**Fig 1:**
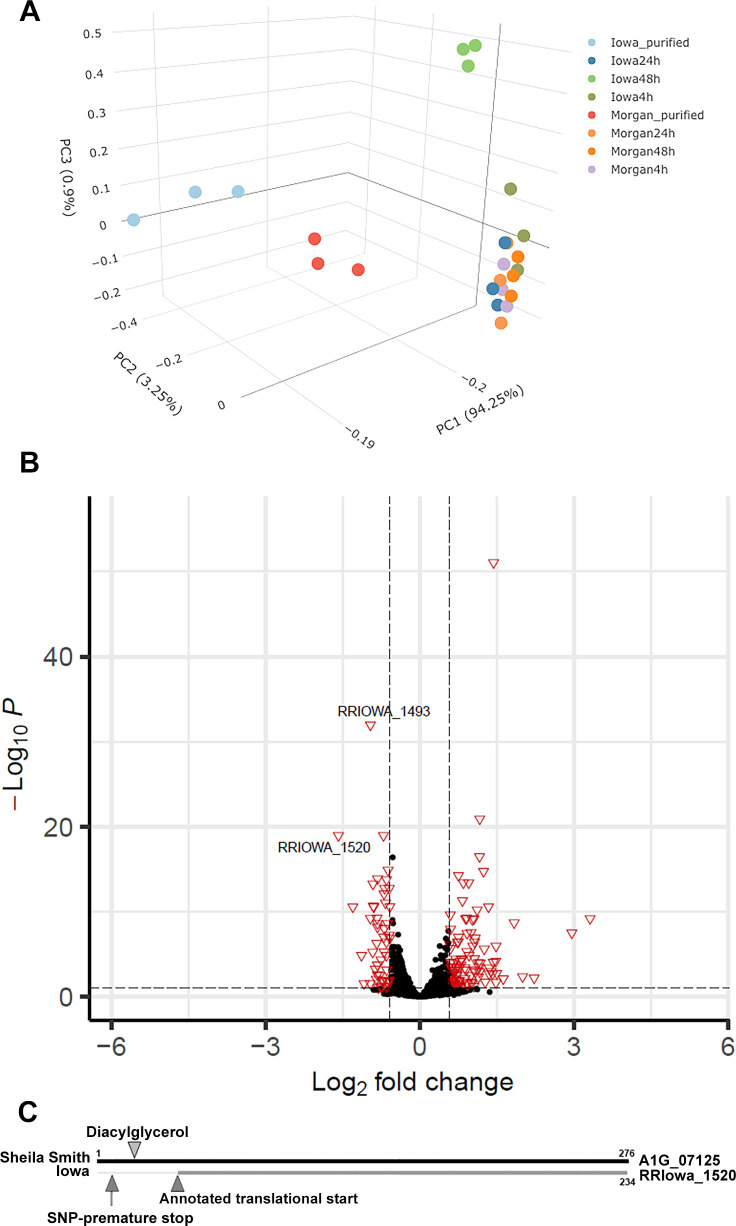
Decreased expression of RRIowa_1520 by *R. rickettsii* Iowa. A. Principal component analysis of RNA isolated from *R*. *rickettsii* Iowa or Morgan strains at 4, 24, and 48 hr post-infection. Purified rickettsiae of each strain were also included in the analysis to account for carry-over mRNA in the infection. B. Volcano plot of RNA-seq data using the 48 hr time point. Horizontal dashed line represents p < 0.05. Vertical dashed lines indicate a positive or negative 1.5-fold change in expression level. C. Schematic of *R*. *rickettsii* Sheila Smith A1G_07125 (RapL) compared to Iowa RRIowa_1520. The predicted site of the signal peptidase 2 cleavage site and the immediate downstream diacylglycerol modified cysteine residue in RapL is identified. The location of the SNP changing the codon to a premature stop in RRIowa_1520 is also identified as is the annotated, predicted translational start site.

Genomic comparisons between Iowa and three virulent strains of *R*. *rickettsii* identified a limited number of SNPs or insertions/deletions unique to the Iowa strain [5,6]. However, none of these genes suggested a role in processing of the autotransporters. We therefore conducted an RNA-seq experiment to identify differences in gene expression that might explain the failure of Iowa to proteolytically process the autotransporters. Because *R*. *rickettsii* Morgan is most closely related to Iowa of the sequenced *R*. *rickettsii* genomes [5], we used the Morgan strain for comparison in this experiment. Although there is no known developmental cycle in rickettsiae, we extracted total RNA for *R*. *rickettsii* infected Vero cells at 4, 24, and 48 hr post-infection. Principal component analysis revealed good congruence of the individual datasets (Fig 1A). While the differentially expressed gene count varied across 4, 24, and 48 hr post-infection *R*. *rickettsii* infected Vero cells, only two genes (RrIowa_1520, RrIowa_1493) showed decreased expression at all timepoints.

The 48 hr time point was chosen for detailed analysis because it showed the greatest number of genes with significant differences in expression levels. A volcano plot showing the genes with a greater than 1.5-fold increase or decrease in expression levels and false discovery rate < 0.1 is depicted in Fig 1B. Notably, the most significantly down-regulated gene in *R*. *rickettsii* Iowa was rOmpA, a large autotransporter containing multiple repeats domains [16] that is not expressed by the Iowa strain [5,6]. The next most significantly down-regulated gene (RRIowa_1520) is annotated as a hypothetical protein. A BLAST search of RRIowa_1520 against other SFG rickettsiae revealed that the Iowa protein is a N-terminally truncated version of *rapL* (WP_012151405.1), which is identical in all other sequenced strains of *R*. *rickettsii* (except HLP-2, which has a single amino acid substitution) and variously annotated by NCBI as a hypothetical protein, peptidase, or alpha/beta hydrolase. Comparison of RRIowa_1520 to all sequenced strains of *R*. *rickettsii* revealed that this truncated form of *rapL* is only found in the three sequenced variants of the Iowa strain. An alignment of RapL from multiple *Rickettsia* spp. shows that it is highly conserved (S2 Fig).

**Fig 2:**
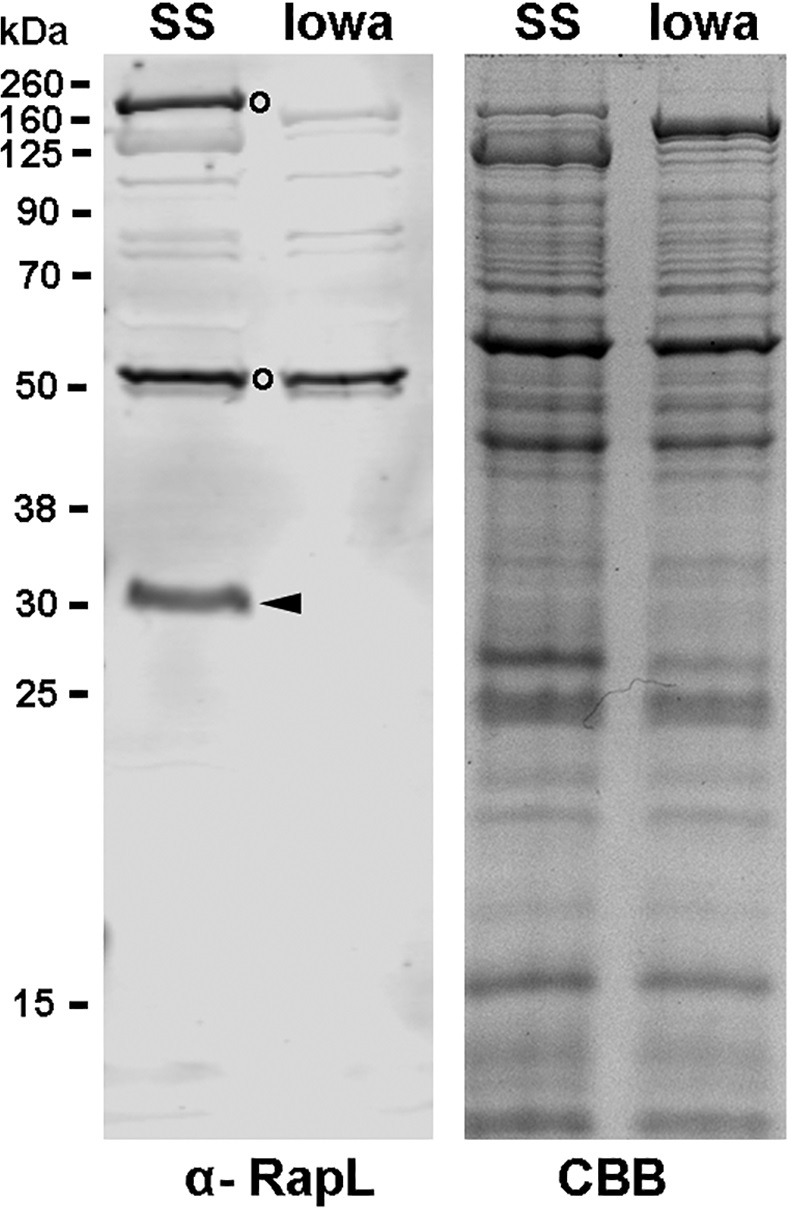
Detection of RapL in *R. rickettsii* Sheila Smith but not Iowa. A 10% SDS-PAGE gel of Sheila Smith (SS) and Iowa was immunoblotted against a polyclonal anti-RapL antibody (α-RapL). Arrowhead indicates the position of RapL from Sheila Smith. No RapL was detected in the Iowa strain. Open circles identify prominent, unidentified cross-reactive bands. A parallel gel stain with Coomassie brilliant blue (CBB) is shown on the right.

A rabbit polyclonal antibody was prepared against RapL and used to probe immunoblots of *R*. *rickettsii* Sheila Smith and Iowa. A band approximating the theoretical Mol Mass of RapL of 30,906 kDa is detected in Sheila Smith but absent from the Iowa strain (Fig 2). The absence of the N-terminal domain from RRIowa_1520 results in undetectable levels of RapL suggesting greatly reduced translation of RrIowa_1520 or instability of the truncated RapL (Fig 2).

Further analysis [17,18] predicts RapL to be a lipoprotein, lipidated at a cysteine residue at amino acid position 19. The Iowa RapL ortholog, RRIowa_1520 contains a premature stop codon. The annotated translational start site of RRIowa_1520 is downstream of the predicted sec secretion signal and lipidated cysteine residue, thus would lack these putative membrane localization signals even if it were to be expressed. A schematic comparing RapL from both the Sheila Smith and Iowa strains is shown in Fig 1C.

### Expression of intact RapL in *R*. *rickettsii* Iowa restores autotransporter processing

**Fig 3:**
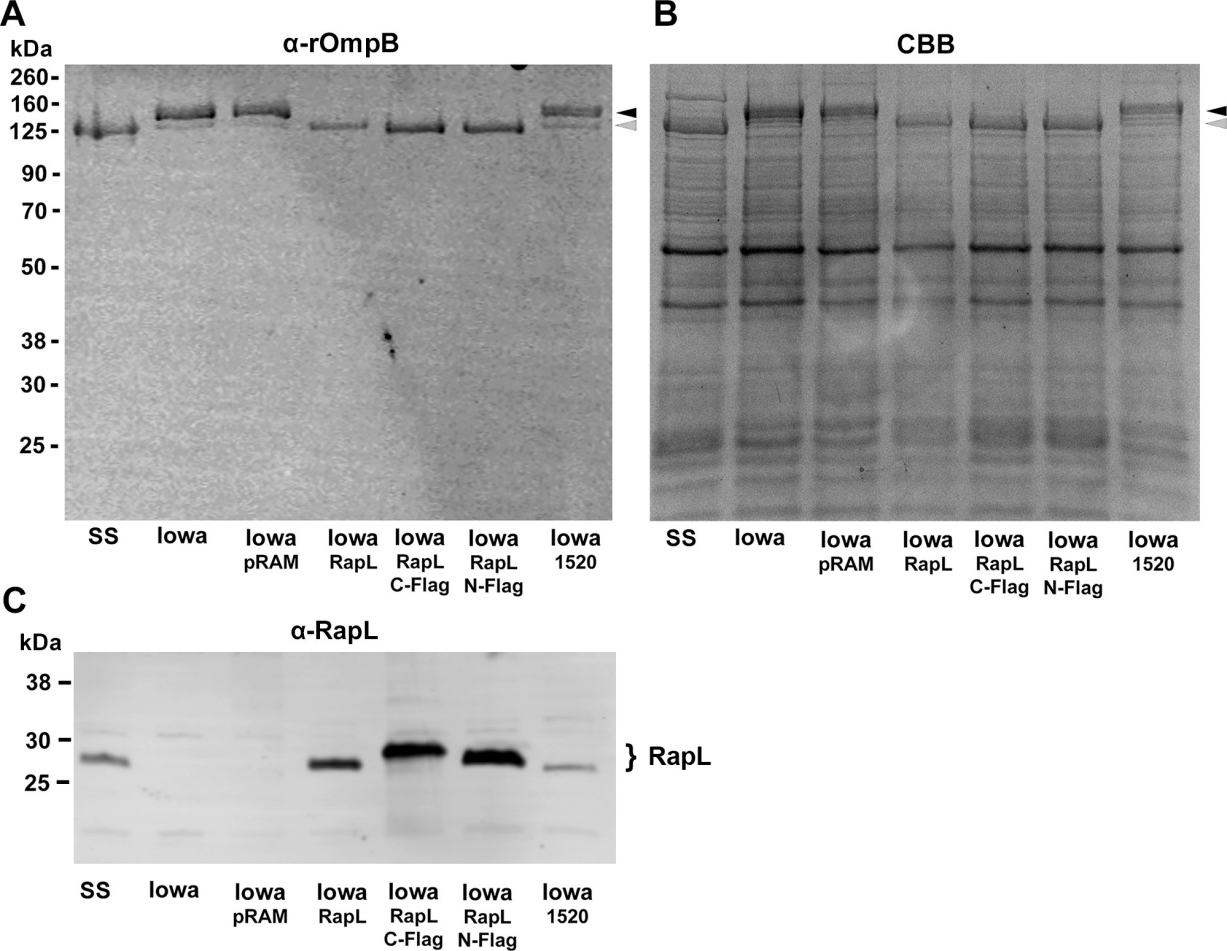
Expression of RapL in *R. rickettsii* Iowa restores appropriate processing of the rOmpB precursor. A. *R*. *rickettsii* Sheila Smith (SS), Iowa, Iowa transformed with the parental plasmid (Iowa-pRAMFC1) (Iowa-pRAM), and Iowa expressing RapL (no Flag tag) (Iowa-RapL), Iowa expressing C-terminally Flag-tagged RapL (Iowa-C-Flag), Iowa expressing N-terminally Flag tagged RapL (Iowa-N-Flag), and Iowa expressing RRIowa_1520 (Iowa-1520) were run on a 10% SDS-PAGE gel and immunoblotted with an anti-rOmpB monoclonal antibody (MAb 13–2) [57]. B. A parallel gel stained with Coomassie brilliant blue (CBB). Black arrowheads indicate the position of the rOmpB precursor and the gray arrows the cleaved, mature rOmpB passenger domain. C. A parallel gel immunoblotted with an anti-RapL antibody. Even when expressed from pRAMFC1, RRIowa_1520 shows reduced translation or instability of the truncated RapL.

RapL from Sheila Smith was cloned into the pRAMFC1 and pRAMFN1 expression vectors for transformation into *R*. *rickettsii* Iowa and examination of effects on autotransporter processing. Three different constructs were generated; RapL alone and with either an N-terminal or C-terminal Flag-tag. Expression of all three constructs of RapL in Iowa resulted in appropriate cleavage of the precursor rOmpB autotransporter into the expected 120 kDa passenger domain fragment (Fig 3). Expression of RRIowa_1520 did not result in processing of rOmpB.

**Fig 4:**
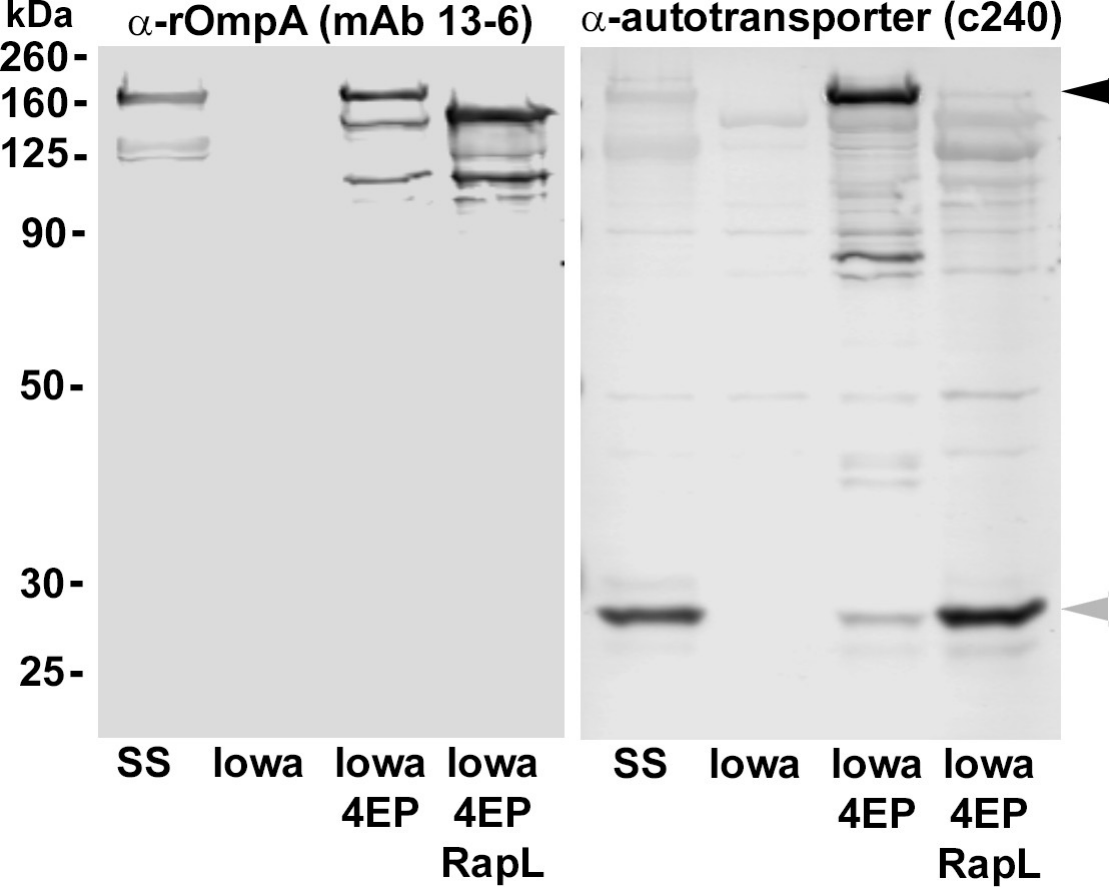
RapL also processes the rOmpA autotransporter. *R*. *rickettsii* Iowa (4 EP small plaque variant) expresses a truncated version of rOmpA lacking four of the thirteen direct repeats of approx. 75 nucleotides each [11]. *R*. *rickettsii* Sheila Smith, Iowa, Iowa 4 EP small plaque variant (Iowa 4EP) and Iowa 4 EP small plaque variant expressing RapL (Iowa 4EP RapL) were run on 10% SDS-PAGE gels and immunoblotted with an anti-rOmpA monoclonal antibody (MAb 13–6) [57] reactive with the passenger domain of rOmpA or with a polyclonal antibody against the β-domain (c240) [11]. Arrowheads on the right indicate the position of the intact Iowa rOmpA precursor (black) and the β-barrel domain (gray).

An early, low passage variant of Iowa that expresses a truncated version of rOmpA lacking four of the thirteen 75 amino acid repeat units [11] was also transformed with pRAMFC1-*rapL* to confirm RapL-dependent cleavage of the precursor rOmpA autotransporter (Fig 4). Proteolytic processing was evidenced by the presence of the passenger domain as well as the autotransporter domain at the predicted sizes by immunoblotting.

**Fig 5:**
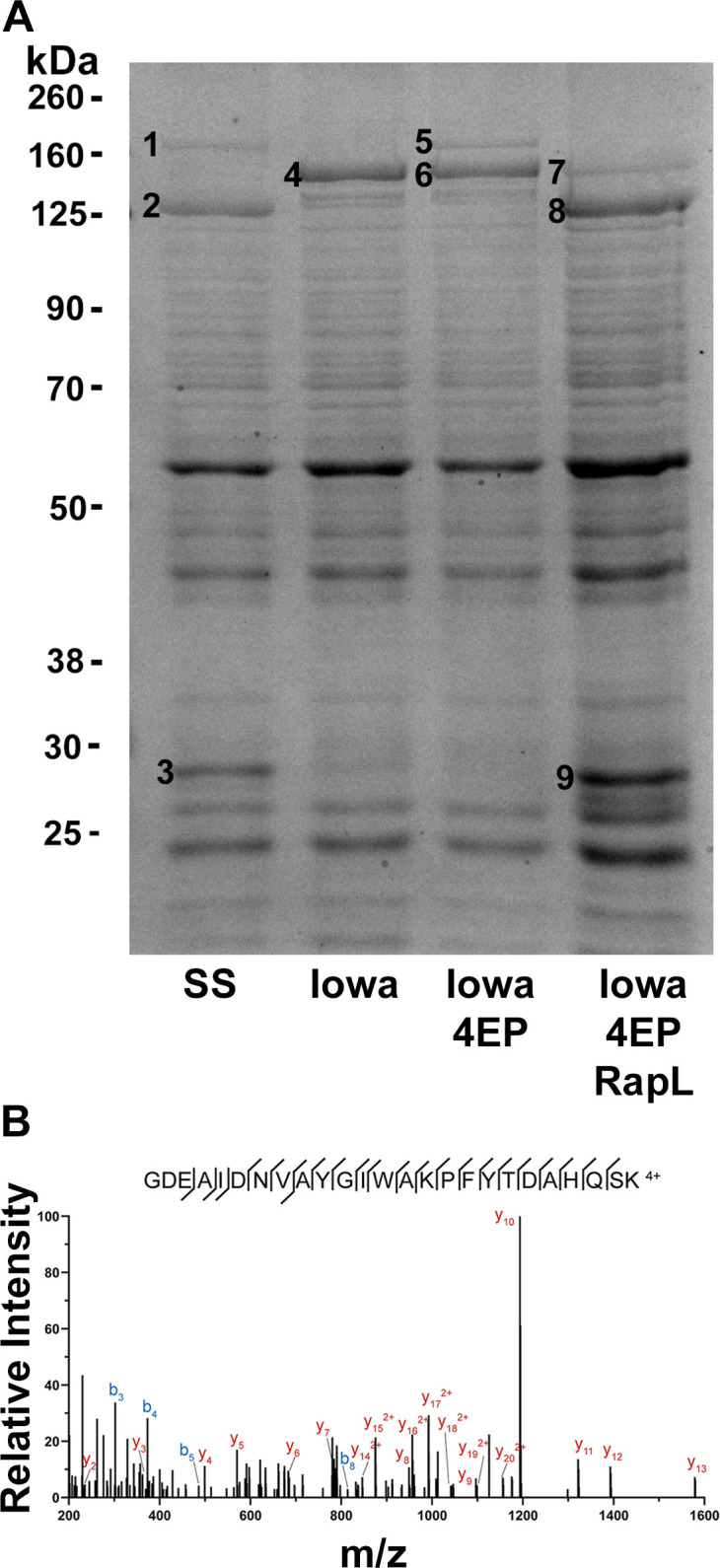
Confirmation of autotransporter species by mass spectrometry. A. *R*. *rickettsii* Sheila Smith (SS), Iowa, Iowa 4 EP small plaque variant (Iowa 4EP) and Iowa 4 EP small plaque variant expressing RapL (Iowa 4EP RapL) were run on 10% SDS-PAGE gels and stained with Coomassie brilliant blue. Numbered bands were excised and subjected to mass spectrometry. Identifications are presented in Table 1. B. Spectra from band 3 identifying the N-terminal peptide of the cleaved rOmpB β-fragment of rOmpB. The identical peptide is found in band 9 from the Iowa 4EP strain expressing RapL-C-Flag. Note that the cleavage site on rOmpB between A1361 and G1362 is not a tryptic cleavage site, thus the presence of this peptide requires the activity of RapL. The full dataset is available at ProteomeXchange Consortium via the PRIDE [55] partner repository with the dataset identifier PXD041355.

The cleavage products of rOmpA and rOmpB were confirmed by SDS-PAGE and mass spectrometry (Fig 5A). Notably, the previously identified N-terminus of the cleaved rOmpB autotransporter domain [9] was confirmed in both the Sheila Smith strain and Iowa 4EP expressing RapL-C-Flag (Fig 5B). In addition, the predicted cleaved autotransporter domains of Sca1 and Sca2, which had not been previously identified, were detected in the MS analysis of the autotransporter domains (bands 3 and 9) in Figs 5A and S1 and Table 1.

**Table 1:** Mass Spectrometry identifications of protein bands from Fig 5.

Band	Predicted	ID (by abundance)^a^
1	rOmpA passenger	rOmpB, dsbA family protein, rOmpA
2	rOmpB passenger	rOmpB
3	rOmpA/B autotransporter	rOmpB, Sca1, rOmpA, Sca2
4	rOmpB precursor	rOmpB
5	rOmpA precursor	rOmpA, rOmpB
6	rOmpB precursor	rOmpB, rOmpA
7	rOmpA passenger	rOmpB, rpoB, rpoC, rOmpA
8	rOmpB passemger	rOmpB
9	rOmpA/B autotransporter	rOmpB, Thioredoxin like, rOmpA, A1G_05640, Sca2

^a^_See S1 Table.

### Modeling of rOmpB amd RapL

**Fig 6:**
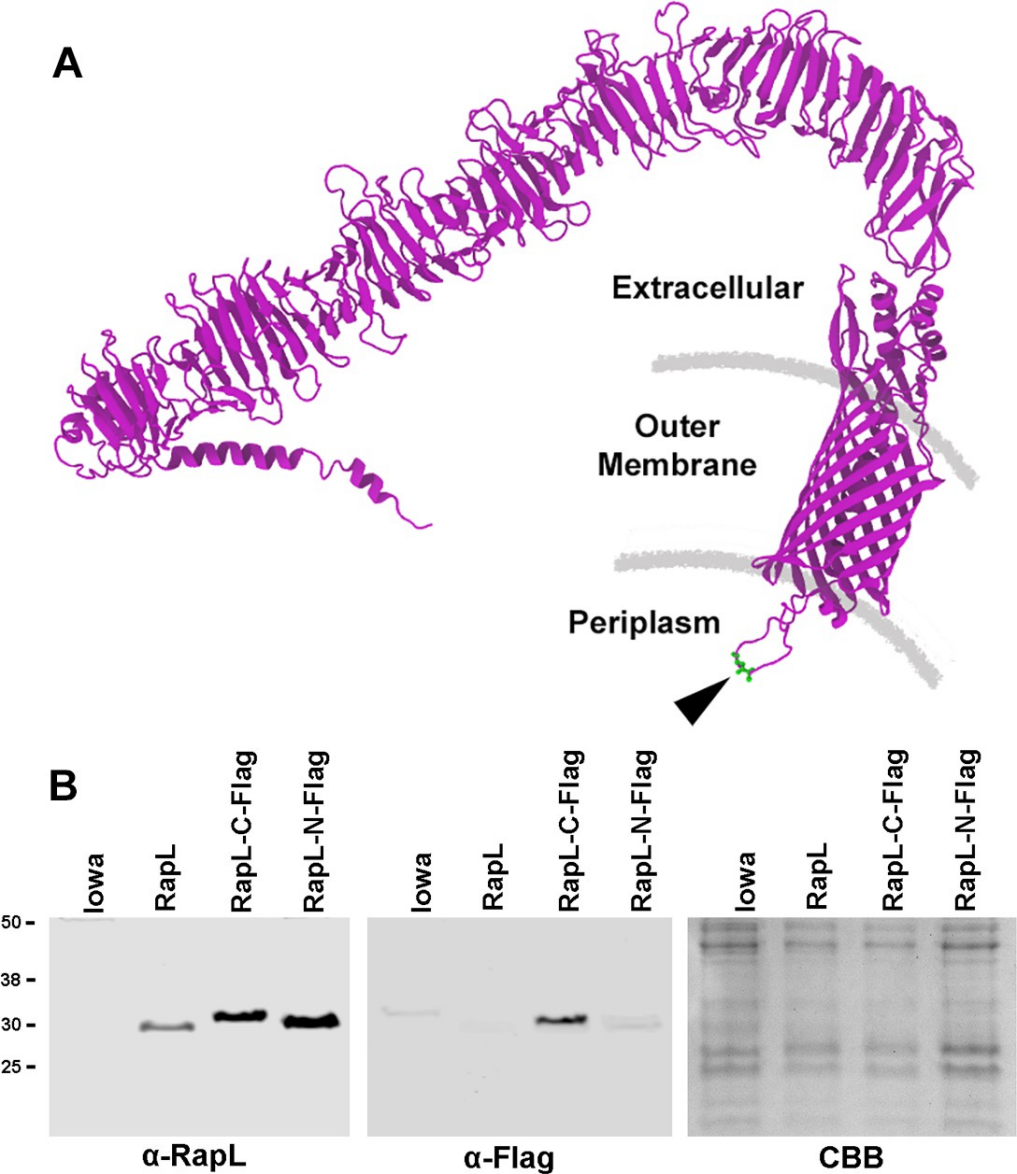
Periplasmic location of RapL. A. Model, predicted by AlphaFold, of the rOmpB autotransporter showing the location of the cleavage site freeing the passenger domain from the β-barrel autotransporter domain. The cleavage site is located near the periplasmic face of the rickettsial outer membrane and the N-terminal three amino acids (GDE) of the cleaved β-domain shown in green as indicated by an arrowhead. The membrane layers are shown as for orientation purposes only and may not reflect actual scale or composition of the layers. B. Cleavage of the secretory signal peptide from RapL. *R*. *rickettsii* Iowa and Iowa expressing RapL (no Flag tag) or C- or N-terminally Flag tagged RapL were immunoblotted against anti-RapL or anti-Flag antibodies. Note that the N-terminally tagged version has the Flag tag cleaved. There is a faint band in the N-terminally flag tagged RapL, which likely represents an unprocessed form, i.e., the N-terminal Flag on the secretory signal is not yet cleaved. A parallel gel stained with Coomassie brilliant blue (CBB) is shown as a loading control.

Modeling of the rOmpB structure indicates that the cleavage site separating the passenger domain from the autotransporter domain is part of a flexible loop-like structure and is exposed in the periplasmic space as the β-barrel extends through the outer membrane (Fig 6A). RapL is predicted to have an N-terminal secretory signal peptide [17]. Comparison of the N- and C-terminal Flag-tagged variants of RapL demonstrate that the N-terminal Flag tag is cleaved following expression in *R*. *rickettsii* suggesting that the N-terminal Flag tag does not interfere with export or cleavage of the secretory signal sequence (Fig 6B). The processing of rOmpB by the expressed RapL-N-Flag is as anticipated for an appropriately localized autotransporter peptidase. Cleavage of signal peptides by signal peptidases 1 or 2 occurs subsequent to translocation into the periplasmic space [19] thus cleavage of the N-Flag provides indirect evidence that RapL is exposed to the periplasmic space.

### Analysis of the predicted RapL peptidase active site

**Fig 7:**
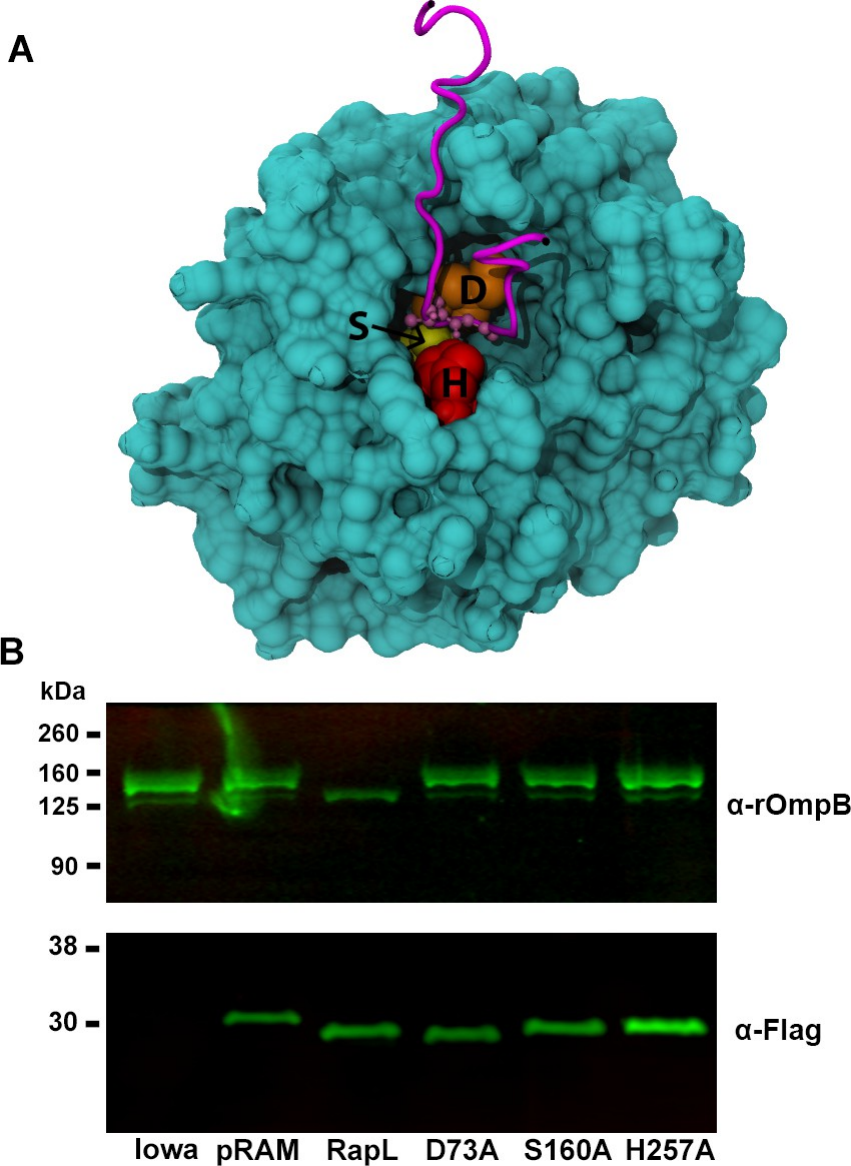
Site directed mutagenesis of amino acid residues comprising a serine protease catalytic triad in RapL inhibits activity. A. Model, predicted by AlphaFold [50], of the cleavage site on rOmpB within a cleft of RapL demonstrating the proximity of amino acids D73, S160, and H257 to the cleavage site. The pLDDT, a per-residue confidence score for the above three residues, are 90.81, 97.38, and 96.94, respectively. The pLDDT values greater than 90 indicate high confidence. B. Immunoblot with anti-rOmpB monoclonal antibody (MAb 13–2) [57] showing that mutation of any of these three amino acids characteristic of a catalytic triad is inhibitory to RapL mediated proteolytic processing of rOmpB. Bottom panel shows expression of the Flag-tagged versions of RapL. Lanes represent the parental Iowa strain, Iowa with the pRAMC1 vector (pRAM) encoding mCherry-Flag, Iowa::pRAMFC1-RapL (RapL), and Iowa::pRAMFC1-RapL encoding each of the site-directed mutants of RapL (D73A, S160A, and H257A).

The structure of RapL was modeled and docked with the rOmpB cleavage site loop to identify a cleft potentially acting as a binding pocket for the autotransporters to promote proteolytic cleavage. The docked model identified five amino acids within 3 A (Fig 7A) of the cleavage site on rOmpB between A1361 and G1362 [9]. Three of the 5 RapL amino acids (D73, S160, and H257) within proximity to the cleavage site are potential components of a serine protease active site catalytic triad [20]. Site-directed mutagenesis of each of these amino acids (D73, S160, and H257) to alanine residues was carried out in an effort to define the active site of the peptidase. The RapL mutant expression plasmids were transformed into *R*. *rickettsii*, Iowa, and examined for effects on rOmpB processing. Each of the mutants of these critical residues of RapL abolished the ability of RapL to proteolytically process the rOmpB autotransporter (Fig 7B) and establishes RapL as a putative serine protease.

### Effect of RapL reconstitution in Iowa on virulence in Guinea pigs

**Fig 8:**
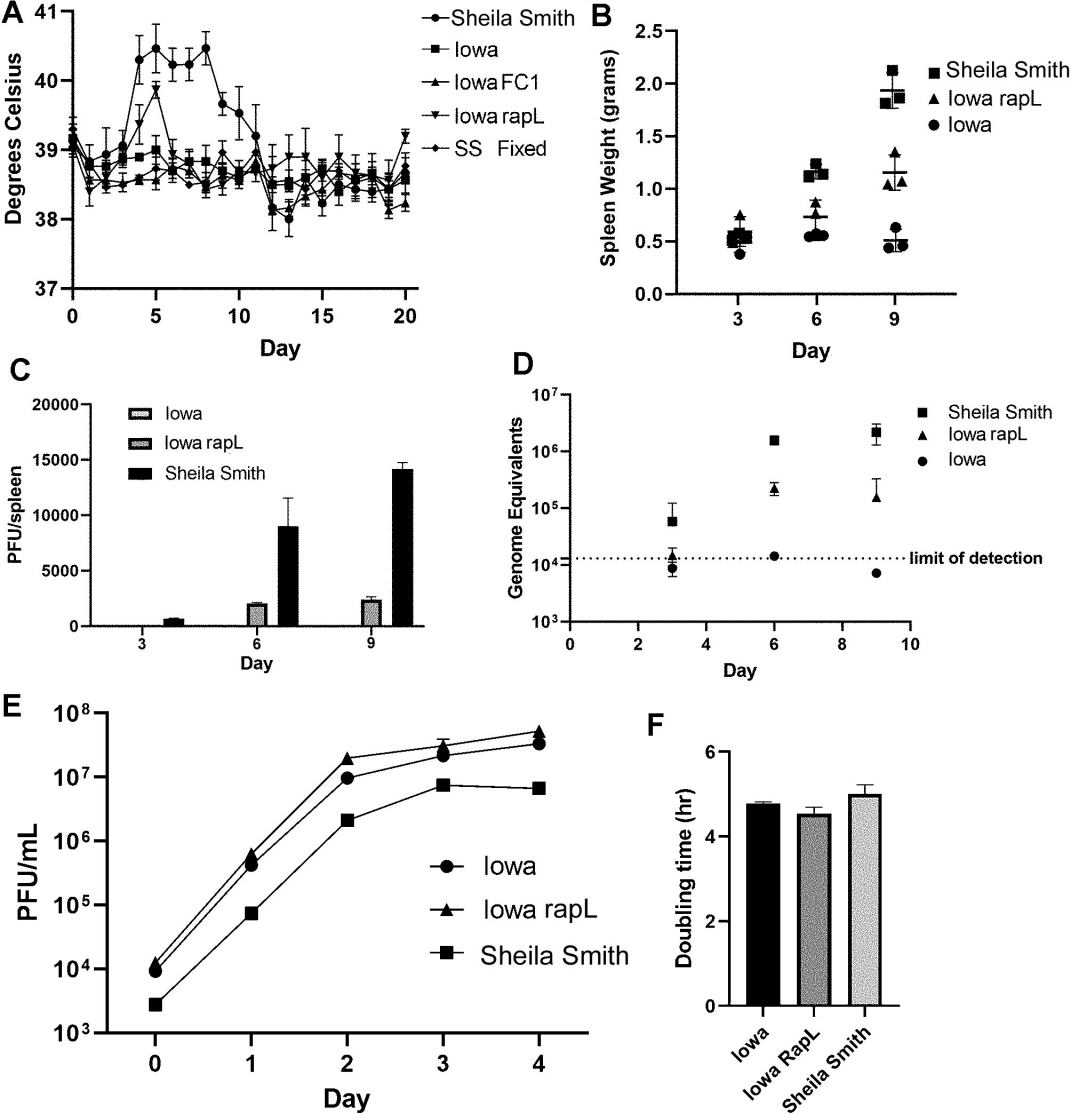
Expression of RapL in *R. rickettsii* Iowa enhances replication *in vivo* and restores a moderate degree of virulence. Animals were infected intradermally with 100 PFU of *R*. *rickettsii* Sheila Smith, Iowa, Iowa with an empty vector (Iowa FC1), or Iowa expressing RapL (Iowa RapL). An equivalent mass of formalin-fixed Sheila Smith (SS Fixed) was inoculated as a control. A. RapL expression causes a mild fever response in *R*. *rickettsii* Iowa. B. RapL expression induces an increase in spleen weights in Guinea pigs. C. Replication of *R*. *rickettsii* Iowa in vivo is enhanced by RapL expression as shown by recovery of viable rickettsiae (C) or genome equivalents (D). Replication rate in Vero cells is not altered by expression of RapL (E, F).

To determine effects of autotransporter processing upon virulence in an animal model system, Guinea pigs were challenged intradermally with 100 PFU of Iowa; Iowa-pRAMFC1 (vector control); Iowa-pRAMF1-rapL (no Flag), and Sheila Smith as a positive control. An equivalent mass of formalin-killed Sheila Smith was inoculated as a negative control. Temperature was monitored for 20 days post-inoculation. *R*. *rickettsii* Iowa expressing RapL showed a modest, but significant, increase in temperature relative to the parental Iowa strain at day 5 post-infection (p < 0.033) (Fig 8A).

At 30 days post-inoculation, sera were taken to determine seroconversion as a sign of infection. Guinea pigs inoculated with the Iowa strain seroconverted as has been observed previously [5,6]; indicating some degree of replication. Interestingly, antibody titers were higher from Guinea pigs infected with *R*. *rickettsii* Iowa expressing RapL than with either the parental Iowa strain or Iowa with the empty plasmid vector suggesting that the RapL expressing strain may replicate to higher titers (S3 Fig). We therefore repeated the experiment to determine rickettsial replication by determining spleen weights, PFUs, and genomic equivalents from spleens on days 3, 6, and 9 post-infection. Spleen weights did not increase in the Iowa infected Guinea pigs but more than tripled in weight by day 9 in the Sheila Smith infected animals and showed an intermediate increase in the Iowa-RapL-infected animals (Fig 8B). Plaque titers of rickettsiae recovered from the spleen showed a similar trend. Only a single plaque was recovered from Iowa infected Guinea pigs from all time points. The Sheila Smith infected animals showed a recovery of 14175 +/- 591 PFU/spleen by day 9 and 2414 +/- 244 PFU/spleen for the Iowa-RapL infected animals. Iowa remained below the limits of detection throughout the experiment (Fig 8C). Genome equivalents were also determined for confirmation of replication with increased sensitivity (Fig 8D). Again, the Iowa strain was at or below the limits of detection by qPCR although the Sheila Smith strain replicated to 2x10^6^ genome equivalents/spleen and Iowa-RapL to approximately 2x10^5^ genome equivalents per spleen. Replication rates in Vero cells were not significantly different between Iowa, Iowa-RapL, and Sheila Smith (Fig 8E and 8F). Collectively, reconstitution of Iowa with rapL promotes increased survival *in vivo* and restores a modest level of virulence associated with appropriate processing of autotransporters.

## Discussion

A failure to correctly process the autotransporter rOmpB was one of the first observed defects in the avirulent Iowa strain of *R*. *rickettsii* [9]. Subsequent genomic comparisons to virulent strains of *R*. *rickettsii* did not identify a candidate protease or molecular basis for processing of rOmpB [5,6]. The genomic comparisons did, however, rule out autocatalytic processing of rOmpB since the sequence of rOmpB from the virulent Morgan strain, which correctly processes rOmpB, is identical to the sequence from the Iowa strain [5]. An RNA-seq experiment was designed to detect missing transcripts not revealed by earlier genomic comparisons [5,6]. A protein variously annotated as an alpha/beta hydrolase, peptidase, or hypothetical protein in all sequenced strains of *R*. *rickettsii*, but truncated in Iowa, was identified, expressed in the Iowa strain, and confirmed as an autotransporter peptidase. Complementation of the Iowa strain with RapL restored appropriate proteolytic processing of all four known autotransporters of *R*. *rickettsii* and restored a modest degree of virulence to the Iowa strain with one day of fever, increased splenomegaly, and increased numbers of rickettsiae recovered from the spleen relative to the parental Iowa strain.

**Fig 9:**
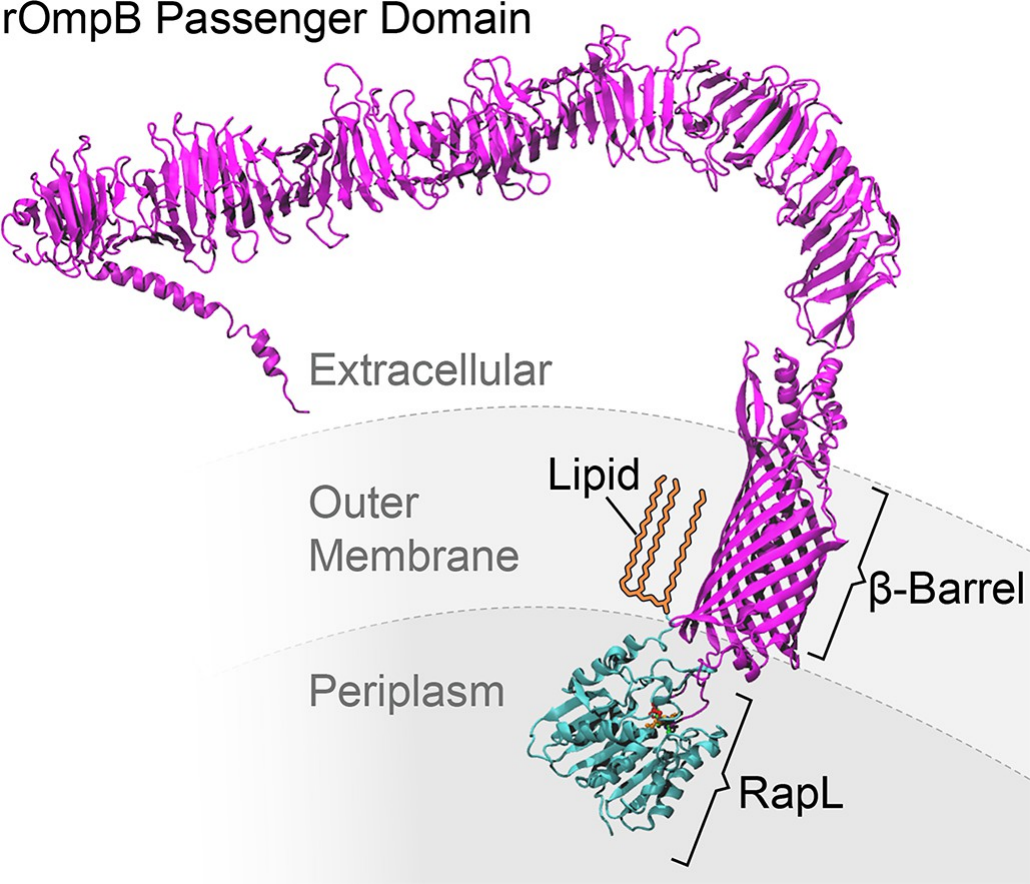
Proposed model of RapL in association with the rOmpB autotransporter. The rOmpB autotransporter is shown in magenta and RapL is in teal. RapL is depicted after cleavage of the N-terminal secretory signal peptide and modification of the N-terminal cysteine residue by lipidation as expected following cleavage by signal peptidase II [27]. The lipid moiety is depicted in yellow. The membrane layers are shown as for orientation only and may not reflect actual scale or composition of the layers.

Once the passenger domain of bacterial autotransporters is exposed on the outer surface of the bacterium, it may remain covalently attached to the β-domain or it may be proteolytically cleaved [10,15]. If cleaved, the passenger domain may be released into the external environment or may remain non-covalently associated with the β-fragment, as does rOmpB of *R*. *rickettsii* [9]. Proteolysis can be autocatalytic [21–24] or may be due to an outer membrane protease [25,26]. In each of these cases, cleavage occurs in the extracellular space. Outer membrane proteases are not well conserved and can be difficult to predict. Efforts to identify the rickettsial autotransporter peptidase based upon similarity searches with known outer membrane proteases had been unsuccessful. Indeed, similarity searches revealed that RapL is largely restricted to the Rickettsiales although more distant similarities were observed in some alpha proteobacteria and *Pseudomonas*. The cleavage site of rOmpB is predicted to be exposed to the periplasm thus it appears that the passenger domain is cleaved with its C-terminal domain extending through the β-barrel into the periplasm. A model of the proposed interaction of RapL with rOmpB is depicted in Fig 9.

Molecular modeling of RapL in association with one of its substrates, rOmpB, indicated five amino acids within 3 Angstroms of the cleavage site on rOmpB. Three of these amino acids, D73, S160, and H257 are potential components of a serine protease catalytic triad [20]. Site-directed mutation of any of these three amino acids inhibited RapL-mediated processing of rOmpB and identifies RapL as a serine protease. Confirmatory studies are ongoing to further characterize RapL enzymatic activities and formally rule out potential indirect effects.

The RapL autotransporter peptidase is itself targeted to the periplasmic space by an N-terminal signal peptide. Signal peptidases are of two types, peptidase I or peptidase II [19,27]. *R*. *rickettsii* expresses both type I and type II signal peptidases [28,29]. RapL appears to be processed by a type II peptidase. Signal peptidase II cleaves lipoproteins following modification of a cysteine residue by a glyceryl moiety after which the signal peptide is cleaved on the N-terminal side of the modified cysteine [27]. Although efforts to identify the lipid moiety are ongoing, rickettsiae express a prolipoprotein diacylglyceryl transferase (NCBI Reference Sequence: WP_012150302.1).

The RNA-seq experiment led to the identification of the long-sought outer membrane peptidase responsible for processing of rickettsial autotransporters. The mutation at coordinate 1202096 of the Iowa genome was noted previously [5] but identified as intergenic due to annotation of the Iowa genome and designation of the Iowa strain as the Reference Strain. This mutation prematurely terminates RapL at amino acid 6 in the Iowa strain; upstream of the predicted lipidation site. Although the Sheila Smith genome was sequenced and deposited in GenBank first, it was not annotated, thus the Iowa genome was annotated for comparison [6] and became the reference strain. The Iowa strain is essentially a laboratory adapted strain, having been passed 271 time through eggs [30]. We have proposed that the virulent Sheila Smith strain be designated as the Reference Strain since it would more accurately reflect the wildtype strains responsible for Rocky Mountain spotted fever.

A previous study had identified a putative rickettsial retropepsin-like aspartic protease in *R*. *conorii* and suggested that it may represent an autotransporter peptidase [31]. However, a number of factors suggest that this protein may not be the authentic autotransporter peptidase. First, the protein in question, rc1339/APRc, is identical in all *R*. *rickettsii* strains (WP_012151442.1), including the Iowa strain, whereas the truncated version of RapL is found only in the three sequenced variants of the Iowa strain [6,11]. A second concern is that evidence of proteolysis was not demonstrated in rickettsiae but through co-expression of rOmpB and rc1339/APRc in *E*. *coli* and *in vitro* analysis. Autotransporters are large, membrane proteins that have frequently been difficult to express in heterologous systems. Furthermore, appropriate processing of rOmpB in the recombinant system was not shown, only the”disappearance” of rOmpB and detection of a his-tagged C-terminal product. The passenger domain of rOmpB was not detected. The role of rc1339/APRc is unclear. Indeed, a more recent study suggests that APRc functions in immune evasion by binding immunoglobulin and promoting resistance to complement-mediated killing thus moonlighting in multiple functions [32]. If rc1339/APRc has a functional role in outer membrane protein maturation, it does not function independently of RapL. In contrast, RapL is sufficient to restore correct processing of the rOmpB and rOmpA precursors in the Iowa strain to the appropriate passenger domain and 32 kDa β-fragments.

The history of the Iowa strain is fragmentary and complex. It was first collected in 1938, prior to the development of plaque assays for rickettsiae. In the first published description, the Iowa strain was described as very mild but, after several passages in eggs, showed increased virulence toward Guinea pigs. After 50 to 100 egg passages the strain reportedly became markedly less virulent and eventually avirulent [30]. An archival vial labeled "Iowa 4 EP", but not dated, was found at the Rocky Mountain Laboratories and the contents plated on Vero cells. Two distinct plaque phenotypes were observed, a large plaque and small plaque. The small plaque variant encoded an almost intact rOmpA; with a deletion of four of the thirteen approx. 75 amino acid repeat units typical of *R*. *rickettsii* rOmpA but an intact autotransporter domain. The large plaque variant has rOmpA truncated within the repeat region and lacks the autotransporter domain. Neither the small nor large plaque variant exhibited virulence in Guinea pigs [11]. Both of these Iowa variants displayed the same truncation of RapL as found in the high egg passages strain of Iowa.

Functions attributed to autotransporters in SFG rickettsiae are multitude. All have at some point been proposed as adhesins [33–38] although certain of these proposals are based upon studies of recombinant *E*. *coli* and have not been confirmed in rickettsiae. Because rickettsiae are obligate intracellular bacteria, they likely have redundant mechanisms of gaining access to the host cell cytoplasm. Knockout of rOmpA, however, had no measurable defect in attachment or entry [11]. Other functions ascribed to rickettsial autotransporters include: rOmpB; associated with avoidance of autophagy [39] or resistance to complement mediated killing [40], and Sca2; responsible for actin-based motility [41,42]. Sca1 of *R*. *felis* is believed to enhance replication in fleas thus promoting potential for transmission [43].

Although *R*. *rickettsii* Sheila Smith and Iowa strains replicate at equivalent rates in Vero cell culture, the virulent Sheila Smith clearly replicates more efficiently *in vivo* using a Guinea pig animal model system. Restoration of RapL in the Iowa strain greatly improves replication/survival *in vivo*. While RapL does not appear to affect growth kinetics in Vero cell culture, it is critical to survival *in vivo*. Presumably, proper processing of one or more of the rickettsial autotransporters is responsible for this enhanced replication *in vivo*. The implication is that a soluble, diffusible passenger domain from at least one of the autotransporters is essential for *in vivo* replication and virulence. At this point, we can only speculate on which autotransporter requires proteolytic processing to promote intracellular survival. rOmpA is not essential for disease in Guinea pigs and it has been suggested that a reason rOmpA is conserved among SFG rickettsiae may be that its role is in the tick host [11]. Similarly, Sca1 from *R*. *felis* appears to be required for survival in fleas [43]. *R*. *rickettsii* Iowa forms actin tails indistinguishable from those of virulent *R*. *rickettsii* [44] and exhibits equivalent size plaques (an indirect measure of actin-based motility) [45] suggesting that proteolytic processing of Sca2 is not essential for actin-based motility. The passenger and autotransporter domains of rOmpB remain non-covalently associated even after proteolytic processing of full-length rOmpB [9]. Interestingly, rOmpB is non-essential for growth in endothelial cells but required for survival in macrophages and colonization of mice [39]. The processing of rOmpB by RapL appears to be important for replication *in vivo* and could provide interesting clues to the function of the passenger domain in rickettsial pathogenesis. The recognition of RapL as a serine protease and virulence determinant further suggests that RapL may be a suitable target for development of therapeutic interventions.

## Materials and methods

### Ethics statement

All animal studies were performed in compliance with a protocol (ASP# 2022-024-E) approved by the Rocky Mountain Laboratories Animal Care and Use committee.

### Cell lines and rickettsiae

Vero76 cells (ATCC CCL-81) were grown in RPMI medium plus 5% heat-inactivated fetal bovine serum (FBS) at 37°C in a 5% CO_2_ atmosphere. *Rickettsia rickettsii* Sheila Smith (CP000848.1), were grown at 34°C in Vero76 cells in M199 medium plus 2% heat-inactivated FBS in a 5% CO_2_ atmosphere. For purification of rickettsiae, infected Vero cells were lysed by Dounce homogenization followed by centrifugation through a 30% Renografin pad. Rickettsiae were washed twice in 250 mM sucrose, and stored in brain heart infusion broth (BHI) at -80°C. Each rickettsial stock was titered for plaque forming units (PFU) by plaque assay. For plaque assays, Vero cells were grown to monolayers in 6-well plates in RPMI 1640 medium with 5% FBS as previously described [41,45,46]. Each rickettsial stock is further characterized for total rickettsial particles by direct rickettsial counts as previously described [47] with the following modifications. Serial dilutions of formalin killed *R*. *rickettsii* purifications were stained with 100ug/ml Acridine Orange (MilliporeSigma, #A3568) and vacuum filtered through black Isopore membrane filters (MilliporeSigma, #GTBP02500). The filters were then mounted on glass slides using immersion oil and observed under 60X magnification using a FITC/TR filter (Omega XF 2010/NS 136). Total numbers of bacteria per milliliter were calculated from the average count of at least 10 random fields of view.

*R*. *rickettsii* strains used here include Sheila Smith large plaque variant (5 egg passages/4 Vero cell passages/1 mouse passage/14 Vero cell passages), Iowa (271 egg passages/1Guinea pig passage/9 Vero cell passages/1 mouse passage/14 Vero cell passages) and Morgan (4 egg passages/7 Vero cell passages/1 mouse passage/14 Vero cell passages).

### Transcriptional profiling

Vero cells in 6-well plates (2/replicate) were infected with *R*. *rickettsii* Morgan or Iowa at a multiplicity of infection (MOI) of 30 for a 4 hr time point and MOI of 1 for 24 and 48 hr time points. Cultures were centrifuged at 2000 RPM for 5 min at room temperature to synchronize infections. All conditions were performed in triplicate. Cultures were incubated at 34°C for 4, 24, or 48 hr prior to harvesting by scraping into Trizol. Purified rickettsiae of each strain were also extracted into Trizol to provide controls accounting for stable RNA in the inoculum. Trizol lysates were extracted following manufacturer’s recommendations (ThermoFisher Scientific, Waltham, MA). RNA containing aqueous phase was extracted using Qiagen AllPrep DNA/RNA 96-well system (Valencia, CA). The RNA yield was determined by spectrophotometry at 260 nm and 280nm and the integrity was assessed using the Agilent 2100 Bioanalyzer using RNA 6000 Pico kit (Agilent Technologies, Santa Clara, Ca). The average RNA Integrity Number (RIN) was 9.2. Ribo-Zero Epidemiology kits (Illumina, Inc, San Deigo, CA) were used to deplete the samples of ribosomal RNAs prior to library preparation with the TruSeq Total RNA-Seq Library Prep Kit (Illumina), starting at the Elute-Frag-Prime step without further modification. Final libraries were assessed on a BioAnalyzer DNA1000 chip (Agilent Technologies, Santa Clara, CA) and quantified using the Kapa Quantification Kit for Illumina Sequencing (Roche, Basel, Switzerland). Paired-end 75 cycle sequencing was completed on a Illumina MiSeq using v3 chemistry generating 14.8 M reads passing filter.

Data files are available under GEO accession number: GSE226832.

### Bioinformatic analysis

The sequencing data was processed using RNA-Seek workflow (https://github.com/OpenOmics/RNA-seek) with default parameters. A custom hybrid reference genome database was built with *Chlorocebus aethiops* (Green Monkey) reference genome (GCA_000409795.2)–background of Vero cells, and *R*. *rickettsii* str. Iowa genome (GCA_000017445.3) using ‘build’ function in RNA-Seek pipeline. Initial data preprocessing included read quality check with FASTQC v0.11.5, screening for sources of contamination (FQScreen v0.9.3, Kraken v2.0.8), removal of adapters and short sequences, and quality trimming with Cutadapt v1.18. Preprocessed reads were then mapped to the hybrid genome reference using STAR v2.7.6a aligner and reads mapping to *Cercopithecus aethiops* and *R*. *rickettsia* genes were quantified using RSEM v1.3.0. The gene quantification data was split between *C*. *aethiops* and *R*. *rickettsia* genes. The *R*. *rickettsia* gene expression data was further processed using iDEP v0.95 [48]. The lowly expressed genes (counts per million < 0.5) were filtered prior to DESeq2 differential expression analyses between Iowa vs. Morgan infected samples at 4h, 24h, 48h and purified samples, respectively. The differentially expressed genes were estimated at adjusted P-value (FDR) threshold of 0.1 and log2(fold change) of 0.5.

### Genomic DNA isolation, PCR, plasmid generation, and construction of *R*. *rickettsii* strains

Rickettsial genomic DNA was isolated by processing individual stock aliquots with the DNeasy Blood and Tissue Kit (Qiagen, 69504). Full-length and truncated forms of *rapL* from Sheila Smith and Iowa respectively were amplified from genomic DNA using primers with BsiWi and BssHII compatible ends(Integrated DNA Technologies) (S2). PCR amplification was performed with Q5 High-Fidelity DNA polymerase (NEB, M0491). Products were isolated using a GeneJet PCR Purification Kit (Thermoscientific, K0702), digested with BsiWi-HF (NEB, R3553S) and BssHII (NEB, R0199S), then re-purified using the GeneJet PCR Purification Kit per manufacturer’s instructions. Plasmids pRAMFN1 and pRAMFC1 were assembled as described previously for pRAMF2 [49], except the *rpsL* promoter replaces the rOmpA promotor for expression of the gene of interest. Vectors were linearized by digesting with BsiWi-HF and BssHII, treated with recombinant shrimp alkaline phosphatase (NEB, M0371S), then run on a 0.8% agarose gel and purified using the GeneJet Gel Extraction Kit (Thermoscientific, K0692). Digested *rapL* PCR products were then ligated into the linearized vector backbones using T4 DNA ligase (NEB, M0202) to generate pRAMF1-rapL NF (no FLAG tag), pRAMFC1-rapL (C-terminal FLAG tag), pRAMFN1-rapL (N-terminal FLAG tag), and pRAMFC1-RrIowa_1520 (C-terminal FLAG tag). Plasmids were transformed into competent *E*. *coli* NEB Stable cells (NEB, C3040H). Sanger sequencing to confirm constructs and PCR products derived from genomic DNA was performed by ACGT Inc. Large scale preparation of plasmids was performed using the GeneJet Plasmid Maxiprep Kit (Thermoscientific, K0492). Purified *R*. *rickettsii* was transformed with plasmids by electroporation as previously described [41]. Clonal transformants were obtained by 4 repetitions of picking isolated plaques and expanding in Vero cell monolayers with M199 containing 200 ng/ml rifampin for PRC verification.

### Molecular modeling

Models of rOmpB and RapL structures, predicted by AlphaFold2 [50], were downloaded from the UniProt database [51]. The predicted structure of RapL (UniProt id A0A0H3AYD9) contained a single domain with high pLDDT scores of 90 or above for most residues. The pLDDT, the predicted local distance difference test, is a per-residue confidence score with values between 0 to 100. Values greater than 90 indicate high confidence, and values below 50 indicate low confidence [50]. The predicted structure of rOmpB (UniProt id Q53047) had multiple domains. Again, the pLDDT scores were above 90 for most residues, indicating a high confidence structure. The confidence in relative positions of the domains, estimated by the Predicted Aligned Error (PAE) scores were mainly less than 5A, except for the relative orientation of the β-barrel autotransporter domain and transporter domains on the outer surface. PAE scores estimate distance errors between pairs of residues. For the subsequent modeling by docking, the uncertainty in relative domain orientation on the outer surface was irrelevant, as the docking took place on the periplasmic side. A 31-residue-long segment, residue 1340–1370, of rOmpB, containing the cleavage site in the middle, was docked with RapL using ClusPro [52]. The top 25 docked models were retained and superimposed on the entire structure of rOmpB. Any docked model creating steric clashes between the full rOmpB and RapL was discarded. Furthermore, models were discarded where the cleavage site of rOmpB was not within 5A of any RapL residues. The remaining 3 models identified possible active residues of RapL, residues within 3A of the cleavage site residues of rOmpB, using VMD [53]. One of the models, where the functional residues of RapL included canonical residues for serine protease, was chosen for mutagenesis studies.

Visual Molecular Dynamics software (https://www.ks.uiuc.edu/Research/vmd/) was used to generate the figures.

### Growth curves for *R*. *rickettsii* strains

For each strain, twelve 25 cm^2^ flasks of confluent Vero cells were infected at an MOI of 0.25 and rocked at room temperature for 30 minutes. 8 ml of M199 + 2% FBS was then added and flasks were incubated at 34°C. At each time point tested, three flasks of each strain were harvested by removal of the medium and scraping the cells into 1 ml of BHI. Samples were added to a microcentrifuge tube containing pre-sterilized glass beads, and host cells disrupted with a Mini Beadbeater (BioSpec Products) for 10 seconds. Samples were divided and stored at -80°C in an isopropanol cooler until quantitation of PFUs.

### SDS-PAGE and Immunoblotting

For purified rickettsia, samples were normalized based upon direct rickettsial counts before pelleting and resuspension in Laemmli sample buffer [54]. Rickettsia-infected Vero 76 cell lysates were generated by adding 100 μl 2x Laemmli buffer per well of a 24-well plate. Samples were inactivated by heating at 99°C for 10 minutes. Samples were run on an SDS-PAGE gel and transferred to a PVDF membrane at 100V for 1 hour and 15 minutes in Towbin transfer buffer containing 20% methanol. Membranes were dried overnight, briefly rehydrated in methanol, rinsed with TBS, and blocked for 1 hour in Odyssey blocking buffer (Li-Cor, 927–50000) at room temperature. Primary antibodies were diluted in blocking buffer with 0.2% Tween 20 while secondaries IRDye 680RD goat anti-rabbit and IRDye 800CW goat anti-mouse (Li-Cor, 926–68071, 926–32210) were diluted 1:10000 in blocking buffer with 0.2% Tween 20 and 0.02% SDS. Blots were then visualized on an Odyssey CLx and analyzed in Image Studio 5.2 (Li-Cor).

### Generation of RapL polyclonal antibodies

Polyclonal antibodies to RapL were generated commercially (Pacific Immunolgy) by immunizing two New Zealand Rabbits with a four-boost series of a synthetic peptide derived from RapL (CVKKQTFPNISHTKGWDK) conjugated to keyhole limpet hemocyanin (KLH). The synthetic peptide derived from RapL was predicted to be antigenic by a proprietary algorithm (Pacific Immunology). A C-terminal cysteine was added to aid in conjugation to the KLH. Sera were collected and antigen affinity purified.

### Mass spectrometry

For the confirmation of cleavage products of autotransporters following RapL expression, purified rickettsiae from strains Sheila Smith, Iowa, Iowa 4 EP small plaque variant (Noriea) and Iowa 4 EP small plaque variant expressing RapL were analyzed by SDS-PAGE and Coomassie brilliant blue staining. Bands of interest were excised for mass spectrometry. Gel bands were diced into 1 mm cubes and washed three times with 25 mM NH_4_HCO_3_, 50% acetonitrile. Proteins were reduced with 10 mM TCEP at 56 °C for 45 min, and then alkylated with 20 mM iodoacetamide at room temperature for 30 min in the dark. Gels were incubated with trypsin in 25 mM NH_4_HCO_3_ solution at 12.5 ng/μL at 37 °C overnight. The resulting peptides were desalted using a C18 ZipTip (Millipore) and analyzed on a Q Exactive plus mass spectrometer (Thermo Fisher Scientific, San Jose, CA, USA) coupled with Easy-nLC 1200 HPLC system. The Q Exactive plus was controlled by Xcalibur software (Thermo Fisher Scientific, Waltham MA, USA). Peptide samples were loaded onto an Acclaim PepMap 100 C18 trap column (75 μm × 20 mm, 3 μm, 100 Å) in 0.1% formic acid and further separated on an Acclaim PepMap RSLC C18 analytical column (75 μm × 250 mm, 2 μm, 100 Å) using an acetonitrile-based gradient (Solvent A: 0% acetonitrile, 0.1% formic acid; Solvent B: 80% acetonitrile, 0.1% formic acid) at a flow rate of 300 nL/min. The gradient was as follows: 0–30 min, 2–40% B; 30–31 min, 30–100% B, 31–32 min, 100% B followed by column wash and re-equilibration to 2% B. The electrospray ionization was carried out with an EASY-Spray Source at a 275°C capillary temperature, 50°C column temperature, and 1.9 kV spray voltage. The mass spectrometer was operated in data-dependent acquisition mode with mass range 375 to 1600 m/z. Full scan resolution was set to 140,000 with AGC target at 3e6 and a maximum fill time of 30 ms. Fragment scan resolution was set to 17,500 with AGC target at 1e5 and a maximum fill time of 70 ms. Normalized collision energy was set to 27. The dynamic exclusion was set with 60 s duration and a repeat count of 1.

Raw data of in-gel digestion samples were analyzed by Proteome Discoverer 2.4. The raw files were searched against *Rickettsia rickettsii* str. ’Sheila Smith’ (NCBI GenBank: CP000848.1, band 1–3), *Rickettsia rickettsii* str. Iowa (NCBI GenBank: CP000766.3, band 4), *Rickettsia rickettsii* str. Iowa 4EP (NCBI GenBank: CP018914.1, band 5–9) and common contaminants (MaxQuant). Search parameters were as follows: Trypsin semi (C-term) with two missed cleavages was set as cleavage enzyme. Carbamidomethylation of Cysteine residue was set as the fixed modification. A total of five variable modifications were allowed per peptide from the following list: oxidation on Methionine, acetylation on protein N-term, pyro-Glutamate conversion on N-term Glutamate, Methionine-loss on protein N-term, and Methionine-loss and acetylation on protein N-term Methionine. The precursor peptide mass tolerance was set to 5 ppm. The fragment ion mass tolerance was set to 0.02 Da. The targeted decoy PSM validator was used for PSM validation. Proteins reported in the result have at least two distinct peptides detected in the analysis.

The mass spectrometry proteomics data have been deposited to the ProteomeXchange Consortium via the PRIDE [55] partner repository with the dataset identifier PXD041355.

### Guinea pig fever curves

Female Hartley Guinea pigs (250–325 g; 5–8 weeks of age) (Charles River Laboratories) were obtained and housed in compliance with a protocol (ASP# 2022-024-E) approved by the Rocky Mountain Laboratories Animal Care and Use committee. To monitor temperatures, transponders (Bio Medic Data Systems, Inc., Seaford, DE) were implanted subcutaneously. 100 PFU were delivered via intradermal injection and temperature recorded daily for 20 days post infection. At 30 days post infection, sera were collected for antibody titration and animals were euthanized.

To determine replication in Guinea pigs, animals were inoculated intradermally with a dose of 100 PFUs each of Sheila Smith, Iowa, and Iowa-pRapL. Spleens were harvested from groups of 3 GP/day on days 3, 6, and 9 post-infection. Individual spleens were weighed and homogenized for determination of PFUs and genome equivalents.

### Estimation of genome equivalents from homogenized spleens

DNA was isolated from homogenized spleens using the DNeasy Blood and Tissue Kit (Qiagen, #69504) according to the manufacturers’ recommendations with the equivalent material of 10 mg of homogenized spleen (calculated from spleen weights). 20 μL qPCR reactions were assembled using 2x TaqMan Fast Advanced Master Mix (Applied Biosystems, #4444557), primers designed to bind to the citrate synthase (*gltA*) gene of *R*. *rickettsii*: 250 nM SFGCS-P (/56-FAM/TGCAATAGCAAGAACCGTAGGCTGGATG/36-TAMSp/), 900 nM SGFCS-F (TCGCAAATGTTCACGGTACTTT) and 900 nM SGFCS-R (TCGTGCATTTCTTTCCATTGT) (Integrated DNA Technologies) [56], and 2 μL of isolated DNA. Reactions were run using StepOnePlus thermocycler (Applied Biosystems) for 40 cycles of 1 second melting at 95°C and 20 seconds of annealing/extension at 60°C. Experiments were run using negative controls without DNA and a dilution curve of a known quantity of genomic DNA isolated from *R*. *rickettsii* Iowa to calculate genome copy numbers.

## Supporting information

S1 Fig A. Schematic of peptides identified in band 3 of Fig 5 showing coverage of the autotransporter domains analyzed. B. N-terminal peptide of Sca2 identified in band 3.
(TIF)

S2 Fig Alignment of RapL from multiple rickettsial species. RapL is highly conserved in spotted fever group rickettsiae with the exception of *R*. *argasii* (KJW04676.1) which encodes a frame shift to create a premature truncation at amino acid 23. Arrowheads indicate position of amino acids making up a putative serine protease catalytic site.
(PDF)

S3 Fig Serological responses of Guinea pigs 30 days following challenge with *R*. *rickettsia* Sheila Smith, Iowa, Iowa-pRAMFC1, Iowa-pRAMFC1-RapL, formalin-fixed Sheila Smith, and K36 buffer. Shown are the Means +/- SEM (n = 3) of ELISA titers against strain Sheila Smith.
(TIF)

S1 Table Proteins identified in each of the bands analyzed by mass spectrometry. (XLSX)

S2 Table Primers used in this study. (XLSX)
