# Supplementary figures and images for: Identification of an autotransporter peptidase of Rickettsia rickettsii responsible for maturation of surface exposed autotransporters
Source: PLoS Pathog. 2023 Jul 31;19(7):e1011527. doi: 10.1371/journal.ppat.1011527 (PMC10414592; doi:10.1371/journal.ppat.1011527)

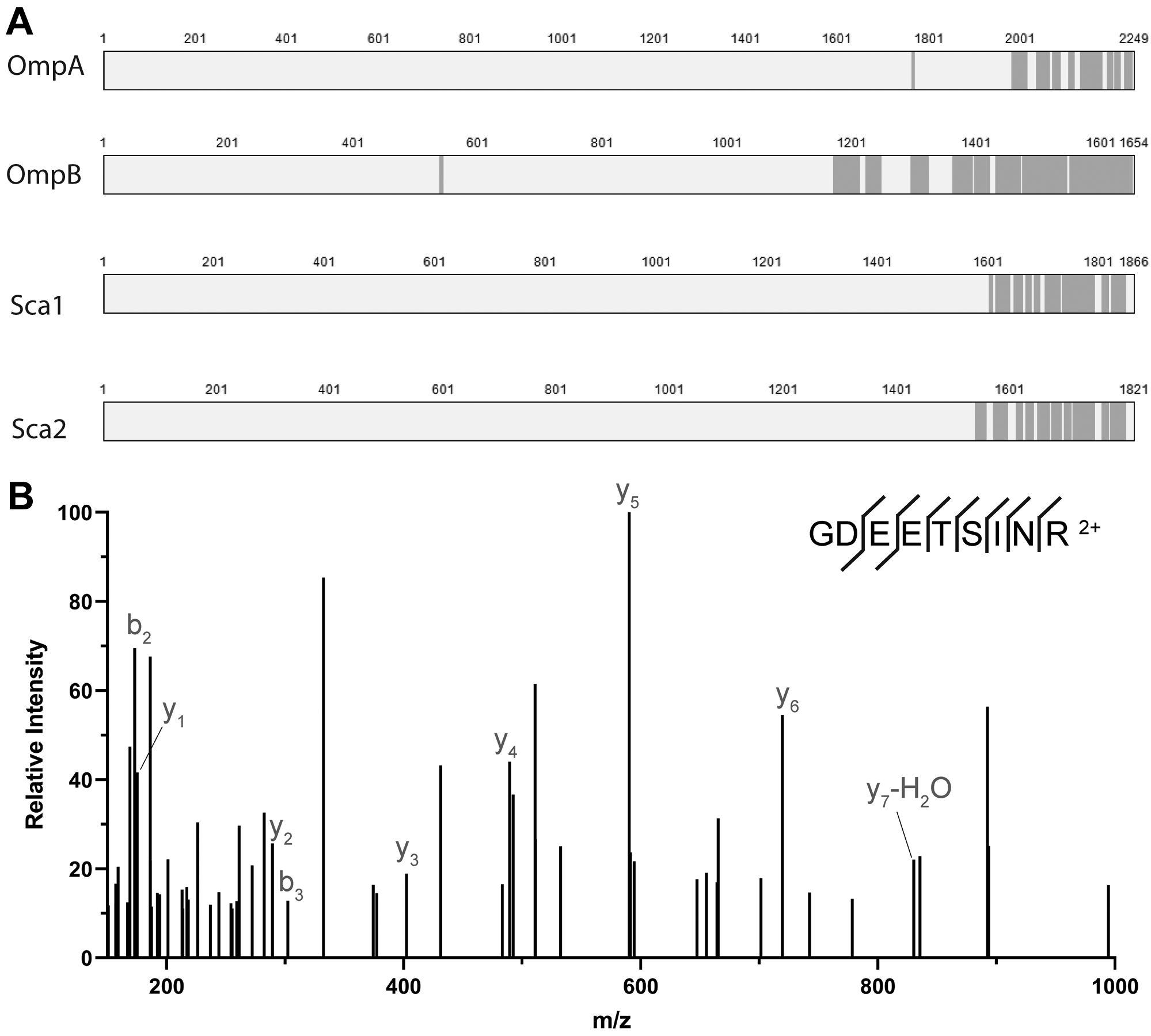

Supplement: S1 Fig — A. Schematic of peptides identified in band 3 of Fig 5 showing coverage of the autotransporter domains analyzed. B. N-terminal peptide of Sca2 identified in band 3. (TIF) [file ppat.1011527.s001.tif]

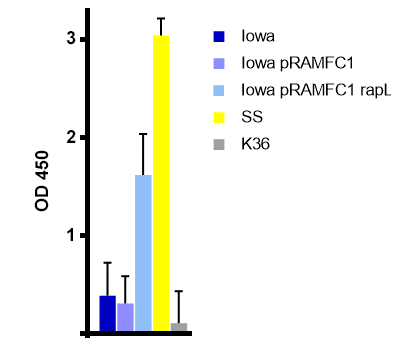

Supplement: S3 Fig — Shown are the Means +/- SEM (n = 3) of ELISA titers against strain Sheila Smith. (TIF) [file ppat.1011527.s003.tif]
