# Supplementary material for: Identification of an autotransporter peptidase of Rickettsia rickettsii responsible for maturation of surface exposed autotransporters
Source: PLoS Pathog. 2023 Jul 31;19(7):e1011527. doi: 10.1371/journal.ppat.1011527 (PMC10414592; doi:10.1371/journal.ppat.1011527)
Supplement: S2 Fig — RapL is highly conserved in spotted fever group rickettsiae with the exception of R. argasii (KJW04676.1) which encodes a frame shift to create a premature truncation at amino acid 23. Arrowheads indicate position of amino acids making up a putative serine protease catalytic site. (PDF) [file ppat.1011527.s002.pdf]

Figure S2: Alignment of RapL orthologs in representative rickettsial species (CLUSTAL O(1.2.4) multiple sequence alignment)

```
Rickettsia rickettsii_Sheila_Smith_rapL                  MKNFIWAIIFLIPIFLSGCSTTHDVARRVKHSQDIATQNNFQMGLVNGGDFTLTTYQRITDRNLPFVFYIEGDGHLADGSANSDNPTPVNPMLLKLATMDKRPNIVYIARPCQYTPLDMN    120
Rickettsia rickettsii_Iowa_RRIOWA_1520                   MKNFI*AIIFLIPIFLSGCSTTHDVARRVKHSQDIATQNNFQMGLVNGGDFTLTTYQRITDRNLPFVFYIEGDGHLADGSANSDNPTPVNPMLLKLATMDKRPNIVYIARPCQYTPLDMN    119
Rickettsia_bellii_strain_AN04_A3306_01190                MKNFI-WIIFLVSVLLSGCSTTHDVASRVKHSQDIAKQNNFQQKLVKGGDFILTTYQRVTDRNQPFVFYIEGDGHLADRGVNSDNPTPVNPMLLKLATIDKRPNVVYIARPCQYTTLEMN    119
Rickettsia_asembonensis_NMRCii_SB78_05065                MKNFIWVIIFLVPILLSSCSTTHDVANRVKHSQDIATQNNFQMQLVNGGDFTLTTYQRITDHNLPFVFYIEGDGHLADGGVNSDNPTPVNPMLLKLATIDKRPNVVYIARPCQYSPLEMN    120
Rickettsia_felis_LSU-lb_JS61_07240                       MKNFIWAIIFLIPIFLSGCSTTHDVASRVKHSQDIATQNNFHMRLVNGGDFTLTTYQRITDHNLPFVFYIEGDGHLADGGVNSDNPTPVNPMLLKLATMDKRPNVVYIARPCQYTPLEMN    120
Rickettsia_aeschlimannii_fig|45262.5.peg.639             MKNFIWAIIFLIPIFLSGCSTTHDIASRVKHSQNIATQNNFQMRLVNGGDFTLTTYQRITNRNLPFVFYIEGDGHLADGSANSDNPTPVNPMLLKLATMDKRPNIVYIARPCQYTPLEIN    120
Rickettsia_philipii_str._364_DRSA_07150                  MKNFIWAIIFLIPIFLSGCSTTHDVARRVKHSQDIATQNNFQMGLVNGGDFTLTTYQRITDRNLPFVFYIEGDGHLADGSANSDNPTPVNPMLLKLATMDKRPNIVYIARPCQYTPLDMN    120
Rickettsia_peacockii_str._Rustic_RPR_05010               MKNFIWAII-LIPIFLSGCSTTHDVARRVKHSQDIATQNNFQMGLVNGGDFTLTTYQRITDRNLPFVFYIEGDGHLADGSANSDNPTPVNPMLLKLATMDQRPNIVYIARPCQYTPLEMN    119
Rickettsia_japonica_strain_M99023_fig|35790.8.peg.1490   MKNFIWAIIFLIPIFLSGCSTTHDVASRVKHSQYIATQNNFQMRLVNGGDFTLTTYQRITDRNLPFVFYIEGDGHLTDGSANSDNPTPVNPMLLKLATMDKRPNIVYIARPCQYTPLEMN    120
Rickettsia_heilongjiangensis_054_Rh054_07045             MKNFIWAIIFLIPIFLSGCSTTHDVASRVKHSQYIATQNNFQMRLVNGGDFTLTTYQRITDRNLPFVFYIEGDGHLTDGSANSDNPTPVNPMLLKLATMDKRPNIVYIARPCQYTPLEMN    120
Rickettsia_montanensis_str._OSU_85-930_MCI_03760         MKNFIWAIIFLIPIFLSGCSTTHDVASRVKHSQDIATQNNFQMRLVNGGDFTLTTYQRITDRNLPFVFYIEGDGHLADGYANSDNPTPVNPMLLKLATMDKRPNIVYIARPCQYTPLEMN    120
Rickettsia_gravesii_BWI-1_fig|1397702.5.peg.1673         MKNFIWAIIFLIPIFLSGCSTIHDVASRVKHSQDIATQNNFQMRLVNGGDFTLTTYQRITDRNLPFVFYIEGDGHLADGSANSDNPTPVNPMLLKLATMDKRPNIVYIARPCQYTPLEMN    120
Rickettsia_africae_ESF-5_RAF_ORF1188                     MKNFIWAIIFLIPIFLSGCSTTHDVARRVKHSQDIATQNNFQMGLVNGGDFTLTTYQRITDRNLPFVFYIEGDGHLADGSANSDNPTPVNPMLLKLATMDKRPNIVYIARPCQYTPLEMN    120
Rickettsia_parkeri_str._Portsmouth_MC1_07155             MKNFIWAIIFLIPIFLSGCSTTHDVARRVKHSQDIATQNNFQMGLVNGGDFTLTTYQRITDRNLPFVFYIEGDGHLTDGSANSDNPTPVNPMLLKLATMDKRPNIVYIARPCQYTPLEMN    120
Rickettsia_sibirica_246_rsib_orf.797                     MKNFIWAIIFLIPIFLSGCSTTHDVARRVKHSQDIATQNNFQMRLVNGGDFTLTTYQRITDRNLPFVFYIEGDGHLADGSANSDNPTPVNPMLLKLATMDKRPNIVYIARPCQYTPLEMN    120
Rickettsia_honei_RB_fig|1175313.3.peg.959                MKNFIWAIIFLIPIFLSGCSTTHDVARRVKHSQDIATQNNFQMRLVNGGDFTLTTYQRITDRNLPFVFYIEGDGHLADGSANSDNPTPVNPMLLKLATMDKRPNIVYIARPCQYTPLEMN    120
Rickettsia_conorii_Malish_7_RC1296                       MKNFIWAIIFLIPIFLSGCSTTHDVARRVKHSQDIATQNNFQMRLVNGGDFTLTTYQRITDRNLPFVFYIEGDGHLADGSANSDNPTPVNPMLLKLATMDKRPNIVYIARPCQYTPLEMN    120
Rickettsia_australis_Cutlack_MC5_01330_correctedstart    -MEKLLRIIFLTSIFLSGCSTIHDVASRVKYSQDIATQNNFQMRLVSGGDFTLTTYQRITASYLPFVFYIEGDGHLADGGVNSDNPTPVNPMLLKLATIDKRPNVVYIARPCQYTPLEMN    119
Rickettsia_akari_str._Hartford_A1C_06475                 -MKKLLYIIFLASIFLSGCSTIHDVASRVKHSQDIATQNNFQMRLVSGGDFTLTTYQRITASDLPFVFYIEGDGHLSDGYVNSENPTPVNPMLLKLATIDKRPNVVYIARPCQYTPLEMN    119
Rickettsia_sp_Tillamook_23_H6P87_01312                   -MKKLLCIIFLTSIFLSGCSTTHDVASRVKHSQDIATQNNFQMRLVNGGDFTLTTYQRITNHNLPFVFYIEGDGHLADGGVNSDNPTPVNPMLLKLATIDKRPNIVYIARPCQYTPLEMS    119
Rickettsia_asiatica_Maytaro1284_RAS_14400_correctedstart -MKKLLCIIFLTSIFLSGCSTTHDVASRVKHSQDIAAQNNFQMQLVNGGDFTLTTYQRITDRNLPFVFYIEGDGHLADGSANSDNPTPVNPMLLKLATMDKRPNIVYIARPCQYTPLEMN    119
Rickettsia_helvetica_C9P9_fig|1144888.4.peg.238          -MKKLLCIIFLTSIFLSGCSTIHDVASRVKHSQDIAAQNNFQMQLVNGGDFTLTTYQRITDRNLPFVFYIEGDGHLADGSANSDNPTPVNPMLLKLATMDKRPNIVYIARPCQYTPLEMN    119
Rickettsia_monacensis_IrR|Munich_RMONA_01190             -MKKLLCIIFLTSIFLSGCSTTNDVASRVKHSQDIATQNNFQMQLVNGGDFTLTTYQRITDRNLPFVFYIEGDGHLADRSANSNNPTPVNPMLLKLATMDKRPNIVYIARPCQYTPLEMN    119
Rickettsia_tamurae_strain_AT-1_fig|334545.3.peg.912      -MKKLLCIIFLTSIFLSGCSTTHDVASRVKHSQDIATQNNFQMQLVNGGDFTLTTYQRITDRNLPFVFYIEGDGHLAGRSANSNNPTPVNPMLLKLATMDKRPNIVYIARPCQYTPLEMN    119
Rickettsia_typhi_TH1527_RTTH1527_03965                   ---MSKFVLLLTS-LLLGCSTTHDVANRIKHAQEIAHQNNFQMKLINGGNFTLTTFQRITANHLPFVFYIEGDGHLTDNGINSNNPTPVNLMLLKLATIDKRSNVVYIARPCQYTPLEMN    116
Rickettsia_prowazekii_Chernikova_M9W_04060               ---MTKFVLLVTSILLLGCSTTHDVASRIKHAQEIATQNNFQMRLINGGDFTLTTFQRITASHLPFVFYIEGDGHLTDNGIHSNNPTPVNLMLLKLATIDKRLNVVYIARPCQYTPLEMN    117
                                                               :: :   :* .*** :*:* *:*::* ** ****:  *:.**:* ***:**:*    ************:.   :*:****** *******:*:* *:*********: *::.

Rickettsia rickettsii_Sheila_Smith_rapL                  PKCISDYWLDKRMGQEVVDSINNVINIINNGQKFSLVGFSGGGGLTILIAAQNSNVKDIITIAGNLDTKKFDKYHNYRGYLVKSLNPINYAKQVNNIPQLHLSGIDDKRVPPFIAALYVK    240
Rickettsia rickettsii_Iowa_RRIOWA_1520                   PKCISDYWLDKRMGQEVVDSINNVINIINNGQKFSLVGFSGGGGLTILIAAQNSNVKDIITIAGNLDTKKFDKYHNYRGYLVKSLNPINYAKQVNNIPQLHLSGIDDKRVPPFIAALYVK    239
Rickettsia_bellii_strain_AN04_A3306_01190                AKCVNDYWLDKRMNHEVVNSINDVINTINNGQKFSLVGFSGGGGLTILIASQNSNVKDIITIAGNLDTEKFDKHHNYRGYLTKSLNPINYAKQISHIPQLHLSGADDKRVPTFIAKLYIN    239
Rickettsia_asembonensis_NMRCii_SB78_05065                PKCVSDYWLDKRMGQEVVDSINNVINIINNGQKFSLVGFSGGGGLTILIAAQNSNVKDIITIAGNLDTKKFDKYHNYRGYLVKSLNPINYAKQVNNIPQLHLSGMDDKRVPPFIAELYVK    240
Rickettsia_felis_LSU-lb_JS61_07240                       PKCVSDYWLDKRMGQEVVDSINNVINIINNGQKFSLVGFSGGGGLTILIAAQNNNVKDIITIAGNLDTEKFDKYHNYRGYLVKSLNPINYAKQVNNIPQLHLSGMNDKRVPPFIAELYVK    240
Rickettsia_aeschlimannii_fig|45262.5.peg.639             SKCISDYWLDKRMGQEVVDSINNVINIINNGQKFSLVGFSGGGGLTILIAAQNSNVKDIITIAGNLDTKKFDKYHNYRGYLVKSLNPINYAKQVNNIPQLHLSGMDDKRVPPFIATLYVK    240
Rickettsia_philipii_str._364_DRSA_07150                  PKCISDYWLDKRMGQEVVDSINNVINIINNGQKFSLVGFSGGGGLTILIAAQNSNVKDIITIAGNLDTKKFDKYHNYRGYLVKSLNPINYAKQVNNIPQLHLSGMDDKRVPPFIAALYVK    240
Rickettsia_peacockii_str._Rustic_RPR_05010               SKCISDYWLDKRMGQEVVDSINNVINIINNGQKFSLVGFSGGGGLTILIAAQNSNVKDIITIAGNLDTKKFDKYHNYRGYLVKSLNPINYAKQVNNIPQLHLSGMDDKRVPPFIAALYVK    239
Rickettsia_japonica_strain_M99023_fig|35790.8.peg.1490   SKCISDYWLDKRMGQEVVDSINNVINIINNGQKFSLVGFSGGGGLTILIAAQNSNVKDIITIAGNLDTKKFDKYHNYRGYLVKSLNPINYAKQVNNIPQLHLSGMDDKRVPPFIAALYLK    240
Rickettsia_heilongjiangensis_054_Rh054_07045             SKCISDYWLDKRMGQEVVDSINNVINIINNGQKFSLVGFSGGGGLTILIAAQNSNVKDIITIAGNLDTKKFDKYHNYRGYLVKSLNPINYAKQVNNIPQLHLSGMDDKRVPPFIAALYLK    240
Rickettsia_montanensis_str._OSU_85-930_MCI_03760         SKCISDYWLDKRMGQEVVDSINNVINIINNGQKFSLVGFSGGGGLTILIAAQNSNVKDIITIAGNLDTKKFDKYHNYRGYLVKSLNPINYAKQVNNTPQLHLSGMDDKRVPPFIAALYVK    240
Rickettsia_gravesii_BWI-1_fig|1397702.5.peg.1673         SKCISDYWLDKRMGQEVVDSINNVINIINNGQKFSLVGFSGGGGLTILIAAQNSNVKDIMTIAGNLDTKKFDKYHNYRGYLVKSLNPINYAKQVNNIPQLHLSGMDDKRVPPFIAALYVK    240
Rickettsia_africae_ESF-5_RAF_ORF1188                     SKCISDYWLDKRMGQEVVDSINNVINIINNGQKFSLVGFSGGGGLTILIAAQNSNVKDIITIAGNLDTKKFDKYHNYRGYLVKSLNPINYAKQINNIPQLHLSGMDDKRVPPFIAALYVK    240
Rickettsia_parkeri_str._Portsmouth_MC1_07155             SKCISDYWLDKRMGQEVVDSINNVINIINNGQKFSLVGFSGGGGLTILIAAQNSNVKDIITIAGNLDTKKFDKYHNYRGYLVKSLNPINYAKQVNNIPQLHLSGMDDKRVPPFIAALYVK    240
Rickettsia_sibirica_246_rsib_orf.797                     SKCISDYWLDKRMGQEVVDSINNVINIINNGQKFSLVGFSGGGGLTILIAAQNSNVKDIITIAGNLDTKKFDKYHNYRGYLVKSLNPINYAKQVNNIPQLHLSGMDDKRVPPFIAALYVK    240
Rickettsia_honei_RB_fig|1175313.3.peg.959                SKCISDYWLDKRMGQEVVDSINNVINIINNGQKFSLVGFSGGGGLTILIAAQNSNVKDIITIAGNLDTKKFDKYHNYRGYLVKSLNPINYAKQVNNIPQLHLSGMDDKRVPPFIAALYVK    240
Rickettsia_conorii_Malish_7_RC1296                       SKCISDYWLDKRMGQEVVDSINNVINIINNGQKFSLVGFSGGGGLTILIAAQNSNVKDIITIAGNLDTKKFDKYHNYRGYLVKSLNPINYAKQVNNIPQLHLSGMDDKRVPPFIAALYVK    240
Rickettsia_australis_Cutlack_MC5_01330_correctedstart    PKCISDYWLDKRMGQEVVDSINNVINIINNGQKFSLVGFSGGGGLTILIAAHNSNVKDIITIAGNLDTKKFDKYHNYRGYLVKSLNPINYAKQVNNIPQLHLSGMDDKRVPPFIAERYVK    239
Rickettsia_akari_str._Hartford_A1C_06475                 PKCISDYWLDKRMGQEVVDSINNVINIINNGQKFSLVGFSGGGGLTILIAAQNNNVKDIITIAGNLDTKKFDKYHNYRGYLVKSLNPINYAKQVNNVPQLHLSGMDDKRVPPFIAELYVQ    239
Rickettsia_sp_Tillamook_23_H6P87_01312                   PKCISDYWLDKRMNQEVVNSINNVINTFNNGQKFSLVGFSGGGGLTILIAAQNSNVKDIITIAGNLDTKKFDKYHNYRGYLVKSLNPINYAKQVNNIPQLHLSGMDDKRVPPFIAELYVK    239
Rickettsia_asiatica_Maytaro1284_RAS_14400_correctedstart SKCISDYWLDKRMGQEVVDSINNVINIINNGQKFSLVGFSGGGGLTILIAAQNSNVKDIITIAGNLDIKKFDKYHNYRGYLVKSLNPINYAKQVNNIPQLHLSGMDDKRVPPFIAELYVK    239
Rickettsia_helvetica_C9P9_fig|1144888.4.peg.238          SKCISDYWLDKRMGQEVVDSINNVINIINNGQKFSLVGFSGGGGLTILIAAQNSNVKDIITIAGNLDIKKFDKYHNYRGYLVKSLNPINYAKQVNNIPQLHLSGMDDKRVPPFIAELYVK    239
Rickettsia_monacensis_IrR|Munich_RMONA_01190             SKCISDYWLDKRMGQEVVDSINNVINIINNGQKFSLVGFSGGGGLTILITAQNSNVKDIITIAGNLDTEKFDKYHNYRGYLVKSLNPINYAKQVNNIPQLHLSGMEDKRVPPFIAELYVK    239
Rickettsia_tamurae_strain_AT-1_fig|334545.3.peg.912      SKCISDYWLDKRMGQEVVDSINNVINIINNGQKFSLVGFSGGGGLTILIAAQNSNVKDIITIAGNLDTEKFDKYHNYRGYLVKSLNPINYAKQVNNIPQLHLSGMEDKRVPPFIAELYVK    239
Rickettsia_typhi_TH1527_RTTH1527_03965                   SKCISDYWVDKRMGQEVVDSINNVINIINNGQKFSLIGFSGGGGLAILIAAQNSNVKDIITIAGNLDTEKFDKYHNYRGYLVKSLNPINYAKQVDNIPQLHLSGMNDKKVPPFIAELYVK    236
Rickettsia_prowazekii_Chernikova_M9W_04060               SKCISNYWLDKRMGQEVVDSINNVINIINNGQKFSLIGFSGGGGLAILIAAQNSDVKDIITIAGNLDTEKFDKYHNYRGYLVKSLNPINYAKQVDNIPQLHLSGMDDKKVPPFIAEQYVK    237
                                                          **:.:**:****.:***:***:*** :********:********:***:::*.:****:******* :****:*******.***********:.: ******* :**:** ***  *::

Rickettsia_rickettsii_Sheila_Smith_rapL                  ASNSPCVKKQTFPNISHTKGWDKVWSNILEIPLICN        276
Rickettsia_rickettsii_Iowa_RRIOWA_1520                   ASNSPCVKKQTFPNISHTKGWDKVWSNILEIPLICN        275
Rickettsia_bellii_strain_AN04_A3306_01190                ASSSNCVKHKVFPNITHTKGWDTVWNSILNIPLNCN        275
Rickettsia_asembonensis_NMRCii_SB78_05065                TSNSPCVKKQTFPNISHAKGWDKIWSNILDTPLICN        276
Rickettsia_felis_LSU-lb_JS61_07240                       TSNSPCVKKQTFPNISHAKGWDKVWSNILDIPLICN        276
Rickettsia_aeschlimannii_fig|45262.5.peg.639             TSNSPCVKKQTFPNISHAKGWDKVWSNILDIPVICN        276
Rickettsia_philipii_str._364_DRSA_07150                  ASNSPCVKKQTFPNISHTKGWDKVWSNILEIPLICN        276
Rickettsia_peacockii_str._Rustic_RPR_05010               TSNSPCVKKQTFPNISHAKGWDKVWSNILDIPLICN        275
Rickettsia_japonica_strain_M99023_fig|35790.8.peg.1490   TSNSPCVKKQTLPNISHAKGWDKVWSNILDIPLICN        276
Rickettsia_heilongjiangensis_054_Rh054_07045             TSNSPCVKKQTLPNISHAKGWDKVWSNILDIPLICN        276
Rickettsia_montanensis_str._OSU_85-930_MCI_03760         TSNSPCVKKQTFPNISHSKGWDKVWSNILDIPLICN        276
Rickettsia_gravesii_BWI-1_fig|1397702.5.peg.1673         TSNSPCVKKQTFPNISHAKGWDKVWSNILDIPLICN        276
Rickettsia_africae_ESF-5_RAF_ORF1188                     TSNSPCVKKQIFPNISHAKGWDKVWSNILDIPLICN        276
Rickettsia_parkeri_str._Portsmouth_MC1_07155             TSNSPCVKKQTFPNISHAKGWDKVWSNILDIPLICN        276
Rickettsia_sibirica_246_rsib_orf.797                     TSNSPCVKKQTFPNISHTKGWDKVWSNILDIPLICN        276
Rickettsia_honei_RB_fig|1175313.3.peg.959                TSNSPCVKKQTFPNISHAKGWDKVWSNILDIPLICN        276
Rickettsia_conorii_Malish_7_RC1296                       TSNSPCVKKQTFPNISHAKGWDKVWSNILDIPLICN        276
Rickettsia_australis_Cutlack_MC5_01330_correctedstart    TSNSPCVKKQTFPNISHAKGWDKVWSNILDIPLICN        275
Rickettsia_akari_str._Hartford_A1C_06475                 TSNSSCVKKQTLPNISHAKGWDKVWANILDIPLICN        275
Rickettsia_sp_Tillamook_23_H6P87_01312                   TSNSPCVKKQTFPNISHAKGWDKIWSNILDIPLICN        275
Rickettsia_asiatica_Maytaro1284_RAS_14400_correctedstart TSNAPCVKKQTFPNISHAKGWDKVWSNILDIPLICN        275
Rickettsia_helvetica_C9P9_fig|1144888.4.peg.238          TSNSPCVKKQTFPNISHAKGWDKVWSNILDIPLICN        275
Rickettsia_monacensis_IrR|Munich_RMONA_01190             TSNSLCVKKQTFPNISHAKGWDKVWSNILYIPLICN        275
Rickettsia_tamurae_strain_AT-1_fig|334545.3.peg.912      TSNSLCVKKQTFPNISHAKGWDKVWSNILYIPLICN        275
Rickettsia_typhi_TH1527_RTTH1527_03965                   ISSSLCVKHHTLPNISHTKGWDKVWSNILDIPLICN        272
Rickettsia_prowazekii_Chernikova_M9W_04060               TSNSLCVKQYTLPNISHTKGWDKVWPHILDIPLICN        273
                                                          *.: ***:  :***:*:****.:*  **  *: **
```
